# Probing Voltage- and Electrolyte-Dependent Monolayer Dynamics with 2D-IR Spectroscopy

**DOI:** 10.1021/jacs.5c14718

**Published:** 2025-10-24

**Authors:** Austin B. Gilbert, Wonjae Jeong, Kyle R. Billings, Aritri Biswas, Matthew J. Ryan, Kijeong Kwac, Minhaeng Cho, Alexei A. Kananenka, Martin T. Zanni

**Affiliations:** ⊥ Department of Chemistry, 5228 University of Wisconsin—Madison, Madison, Wisconsin 53706, United States; ‡ Department of Physics and Astronomy, 5972 University of Delaware, Newark, Delaware 19716, United States; § Center for Molecular Spectroscopy and Dynamics, 364806 Institute for Basic Science (IBS), Seoul 02841, Republic of Korea; ∥ Department of Chemistry, Korea University, Seoul 02841, Republic of Korea

## Abstract

Little is known about structural dynamics at electrode surfaces under applied potential. Here, we report 2D-IR spectra of a 4-mercaptobenzonitrile monolayer on a gold electrode, revealing chemical exchange between two subensembles on sub- to tens-of-picoseconds time scales. Dynamics are measured in 100 mM MgCl_2_, LiCl, and KCl at −200 and +300 mV vs Ag/AgCl. At +300 mV, all electrolytes exhibited ≥48 ps exchange, while at −200 mV, the slower exchange component ranged from ≥33 to ≥43 ps. The dynamics correlate with ion concentration, according to radial distribution functions calculated from molecular dynamics simulations, suggesting that local ion densities, regardless of valency or sign, slow dynamics. Results show solvation, electric double layer formation, and monolayer reorientation. This work reveals how electrolyte composition modulates molecular reorientation and hydrogen bonding at functionalized electrodes.

## Graphical abstract

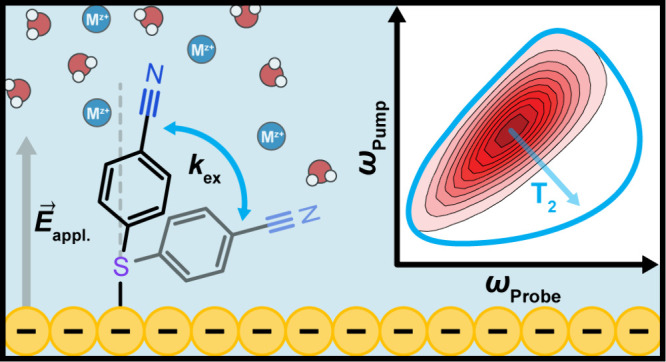

Electrode–electrolyte interfaces govern electrocatalysis, energy storage, and sensing.^1,2^ Central to these processes is the electrolyte, which balances charge and defines the electric double layer (EDL). When voltage is applied, ions rearrange, confining electric fields to nanometer scales.^3–9^ Ion accumulation modifies solvation, hydrogen bonding, and the local environment at the surface.^10–31^ When molecules are tethered to electrodes, such as redox catalysts, the applied potential modulates electronic and structural properties. Electrolyte identity alters ion distributions, tuning solvation and hydrogen bonding.^1^

Despite their importance, experiments that probe the dynamics of the EDL and monolayer under an applied voltage are limited. The EDL is confined to a few nanometers, and monolayers are only one molecule thick, thus requiring very sensitive experiments. Surface-enhanced infrared absorption spectroscopy (SEIRAS) and sum frequency generation (SFG) offer monolayer structural sensitivity, but lack temporal resolution.^6,31–38^ Terahertz spectroscopy has further provided complementary insight into ion hydration and collective water dynamics within the electric double layer, but lacks molecular specificity.^39,40^ A two-dimensional (2D) version of SFG called two-dimensional heterodyne-detected vibrational sum-frequency generation (2D-HD-VSFG), has monolayer sensitivity and can measure dynamics, but has not been performed under an externally applied electrode potential.^41–45^ Currently, the most detailed insights on voltage-dependent dynamics are from molecular dynamics (MD) simulations, which provide an atomistic detail of electrode–electrolyte systems.^19,20,46–50^ Thus, dynamics at electrode surface is an outstanding scientific problem.

Using plasmonic enhancement, it is possible to apply standard 2D-Infrared (2D-IR) spectroscopy, a technique for studying dynamics in bulk liquids, to the surfaces of electrodes under an applied potential.^51–53^ The technique is termed surface-enhanced (SE) 2D-IR. We previously reported SE-2D-IR of 4-mercaptobenzonitrile (4-MBN) monolayers on a gold electrode in ultrapure water. 4-MBN forms self-assembled monolayers (SAMs) on gold (Au) and features a nitrile group (Figure 1) that is sensitive to both electric fields and hydrogen bonding.^31,54–56^ We observed two subpopulations undergoing dynamic chemical exchange. The rate of exchange between the two subpopulations was voltage dependent, ranging from 25 to ≥ 63 ps. The subpopulations were previously assigned to hydrogen bonded and non-hydrogen bonded 4-MBN molecules.^31^ Follow-up simulations by Kwac et al. found that hydration was accompanied by an orientational change of 4-MBN and assigned the 10s-of-ps dynamics to structural dynamics of the monolayer molecules, which depends on the applied potential.^57^

**Figure 1:**
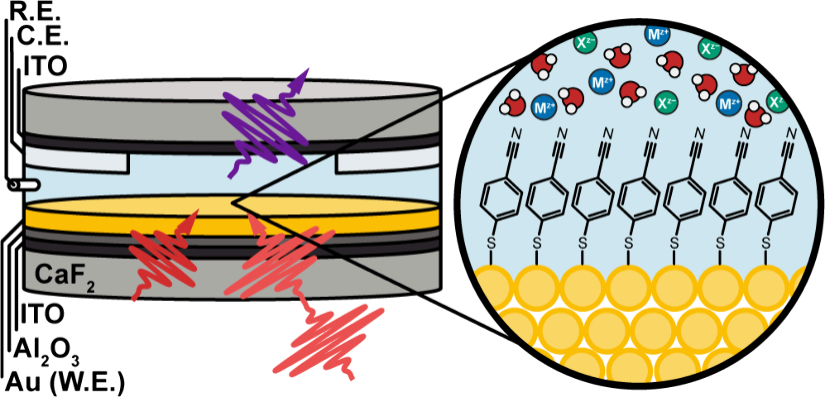
Schematic of 2D-IR spectroelectrochemical cell. The CaF_2_ substrate is deposited with thin layers of ITO, Al_2_O_3_, and plasmonic Au. W.E., C.E., and R.E. stand for working, counter, and reference electrode, respectively. Shown in the zoomed inset is a schematic of 4-MBN SAM and representative EDL structure.

In the absence of a supporting electrolyte, our prior water-only study probed an interface governed by the salt-free Poisson–Boltzmann (Gouy–Chapman) limit, resulting in minimal double-layer formation.^58^ Here, we extend this study to potassium chloride (KCl), lithium chloride (LiCl), and magnesium chloride (MgCl_2_) electrolytes, a subset of the Hofmeister series of salts for which solvation and EDL behavior will vary due to differences in ionic radii, charge densities, and polarizability.^59–61^ These ionic properties determine hydration structure and strength, affecting how water interacts with both the electrode and tethered molecules. These hydration characteristics shape hydrogen bond networks at the interface, influencing the frequency and time scale of reorientational dynamics for surface-bound probes. We find that the chemical exchange dynamics between the two subpopulations depend on both electrolyte and applied voltage, demonstrating that electrolyte composition modulates structural dynamics at the electrode. The experiments reported here provide the first dynamic 2D-IR measurements of ions and molecules at an electrode surface.

As described previously, we used a custom-built spectroelectrochemical cell enabling transmission-mode SE-2D-IR (Figure 1).^31^ The working electrode (W.E.) comprises nonconductive plasmonic gold and a thin conductive layer separated by an insulating layer, all designed to complete the circuit while minimizing background and eliminating Fano line shape distortions. A platinum counter electrode (C.E.) and silver/silver chloride (Ag/AgCl) reference electrode (R.E.) complete the three-electrode configuration.^31^ Au electrodes were soaked overnight in 30 mM 4-MBN in ethanol, rinsed, and dried under nitrogen (N_2_). Additional details on electrode preparation can be found in Supporting Information. Experiments were conducted in 100 mM aqueous electrolyte (KCl, LiCl, or MgCl_2_). In this regime, the EDL is described by the Gouy–Chapman-Stern framework.^1^ 2D-IR spectra were collected across t_2_ delays from 0 to 15 ps at −200 mV and +300 mV vs Ag/AgCl, conditions within a conservative electrochemical window.

Figure 2a–d shows 2D-IR spectra for MgCl_2_ at −200 mV. A cross-peak emerges below the diagonal with increasing delay, appearing as broadening shoulder. The cross-peak is apparent in horizontal cuts through the 2D-IR spectra normalized to the diagonal intensity to remove population relaxation (Figure 2e). Previous control experiments and simulations support that the observed cross-peak arises from exchange between two overlapping subpopulations centered at 2222 and 2228 cm^–1^ (Figure 2f) on a picosecond time scale.^31^

**Figure 2:**
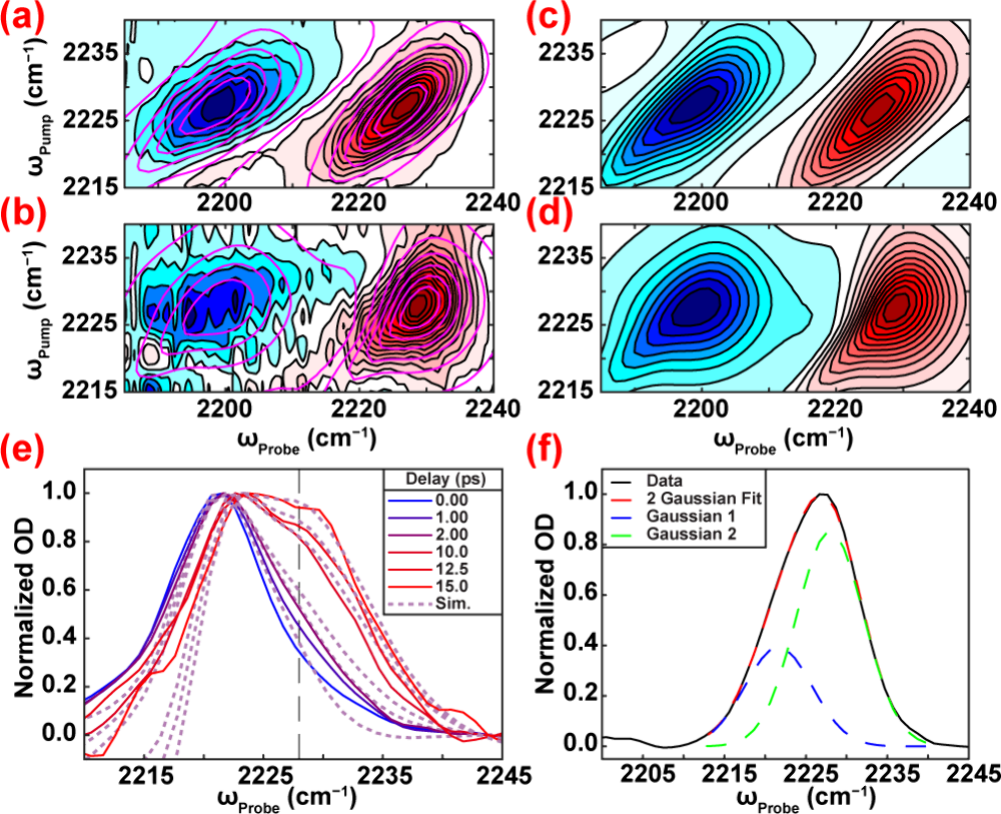
Experimental 2D-IR spectra of 4-MBN at −200 mV vs Ag/AgCl in 100 mM MgCl_2_ at (a) 0 ps and (b) 15 ps delay (overlaid simulated 2D contours) and simulated 2D-IR spectra at (c) 0 ps and (d) 15 ps delay. (e) Horizontal pump slices from 15 ps spectrum of 4-MBN in 100 mM MgCl_2_ at ω_pump_ = 2222 cm^–1^ (overlaid simulated slices) (f) Normalized diagonal slice from 0 ps spectrum of 4-MBN in 100 mM MgCl_2_ fit to a sum of two gaussians.

To quantify rates, we simulated the 2D-IR spectra from 0–15 ps (Figure 2a–d). Simulations followed our previously reported workflow, in which the t_0_ diagonal slice was fit to the sum of two Gaussian functions to extract the center frequencies and relative populations (Figures S7–S8 in Supporting Information). These experimental parameters initialize a Kubo line shape model containing multiple frequency-frequency correlation function (FFCF) components to reproduce the early time broadening and a single chemical exchange rate constant governing long-time cross-peak growth.^31,62^ This approach yields physically meaningful exchange rates directly comparable across electrolytes (Table 1). The simulated spectra match the experiments and reported uncertainties reflect the range of chemical exchange rates that preserve good agreement between experimental and simulated data (Figures S9–S20). These intervals define lower-bound estimates given the 15 ps temporal window. Additional simulation details can be found in Supporting Information.

**Table 1:** Chemical Exchange Rates Extracted from Simulated 2D-IR Spectra^a^

Salt (Voltage)	Chemical Exchange Rate (×10^2^ ps^–1^)^b^
MgCl_2_ (−200 mV)	2.5 ± 0.1 (≥38 ps)
LiCl (−200 mV)	2.9 ± 0.1 (≥33 ps)
KCl (−200 mV)	2.0 ± 0.3 (≥43 ps)
MgCl_2_, LiCl, KCl (+300 mV)	2.0 ± 0.1 (≥48 ps)

a Additional simulation parameters given in Supporting Information.

b Parenthetical time scales reflect the lower-limit estimates (≥) obtained from the upper-bound exchange rates; time scale errors are asymmetric due to nonlinear rate−time conversion.

The MgCl_2_ results are shown again in Figure 3, alongside similar slices for experiments and simulations on LiCl and KCl at −200 mV. It is readily apparent that the cross-peak intensity is similar for MgCl_2_ and LiCl, but the cross-peak is much smaller at 15 ps for KCl, indicating slower chemical exchange kinetics. Cross-peak intensity vs delay is shown for each salt (Figure 3d–f). Kinetics evolve with a subpicosecond rise and a slower tens-of-ps component. Complete parameters used for the simulations are given in Supporting Information. At −200 mV, MgCl_2_ and LiCl exchange at 2.5 ± 0.1 × 10^–2^ and 2.9 ± 0.1 × 10^–2^ ps^–1^, respectively, while KCl is slower at 2.0 ± 0.3 × 10^–2^ ps^–1^ (Table 1).

**Figure 3:**
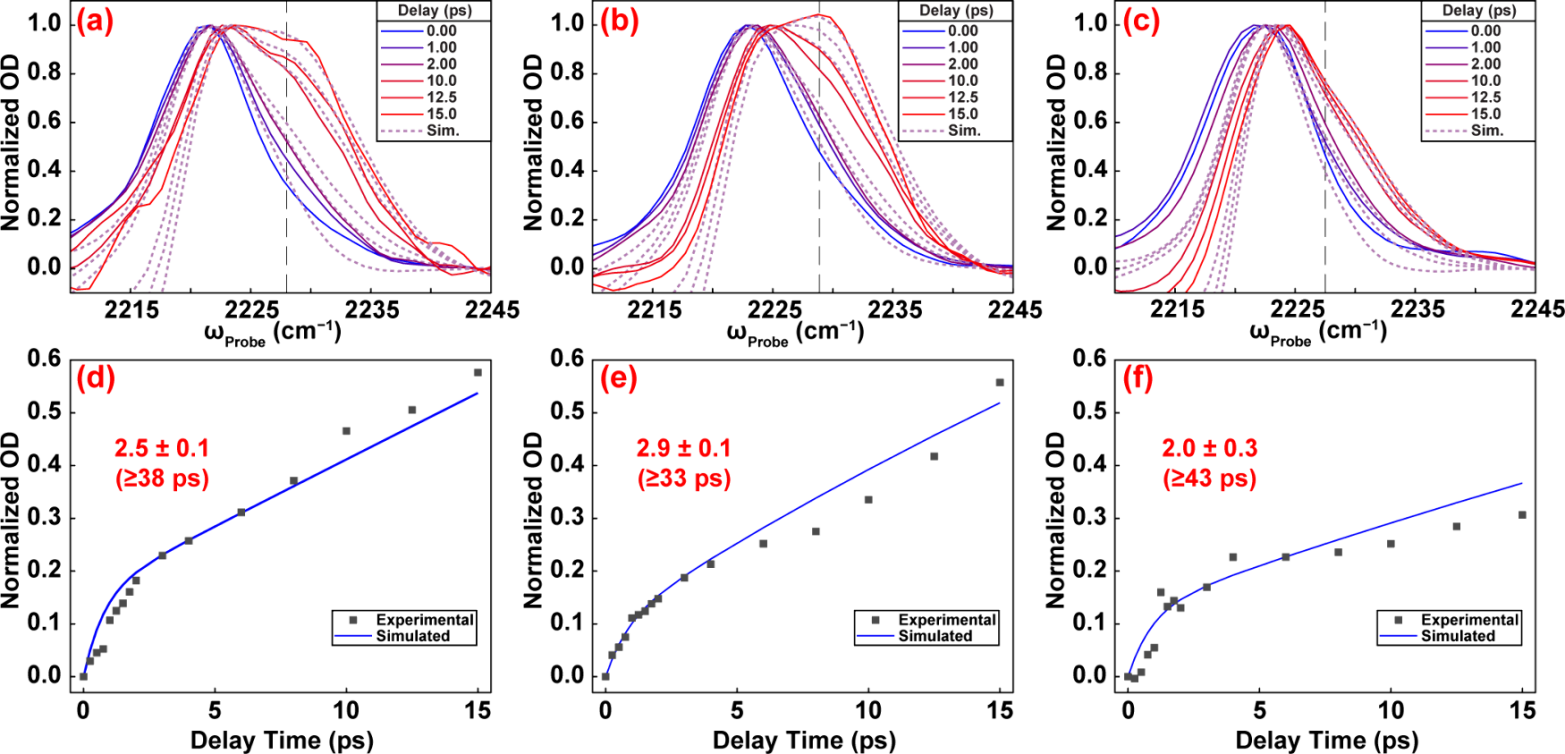
Comparison of MgCl_2_, LiCl, and KCl spectra and kinetics at −200 mV vs Ag/AgCl. Experimental pump slices (overlaid with simulated slices) from 15 ps spectra of 4-MBN in 100 mM (a) MgCl_2_, (b) LiCl, and (c) KCl. Kinetic traces of cross-peak evolution in experimental and simulated data for (d) MgCl_2_, (e) LiCl, and (f) KCl samples.

Shown in Figure 4 are slices and kinetics for these three salts at a positive potential of +300 mV (complete data sets in Supporting Information). Unlike at negative potential, the growth of the cross-peak is nearly identical for all three salts, and the kinetics are slow. At +300 mV, the data are all well-simulated using a chemical exchange rate of 2.0 ± 0.1 × 10^–2^ ps^–1^ (Table 1). We do not resolve differences in the subpicosecond component of the kinetics, which includes contributions from both spectral diffusion and chemical exchange, possibly because our time resolution is roughly 200 fs. For clarity, the extracted chemical exchange rates are labeled on the kinetic traces in Figure 3d–f and Figure 4d–f to enable direct visual comparison across electrolytes.

**Figure 4:**
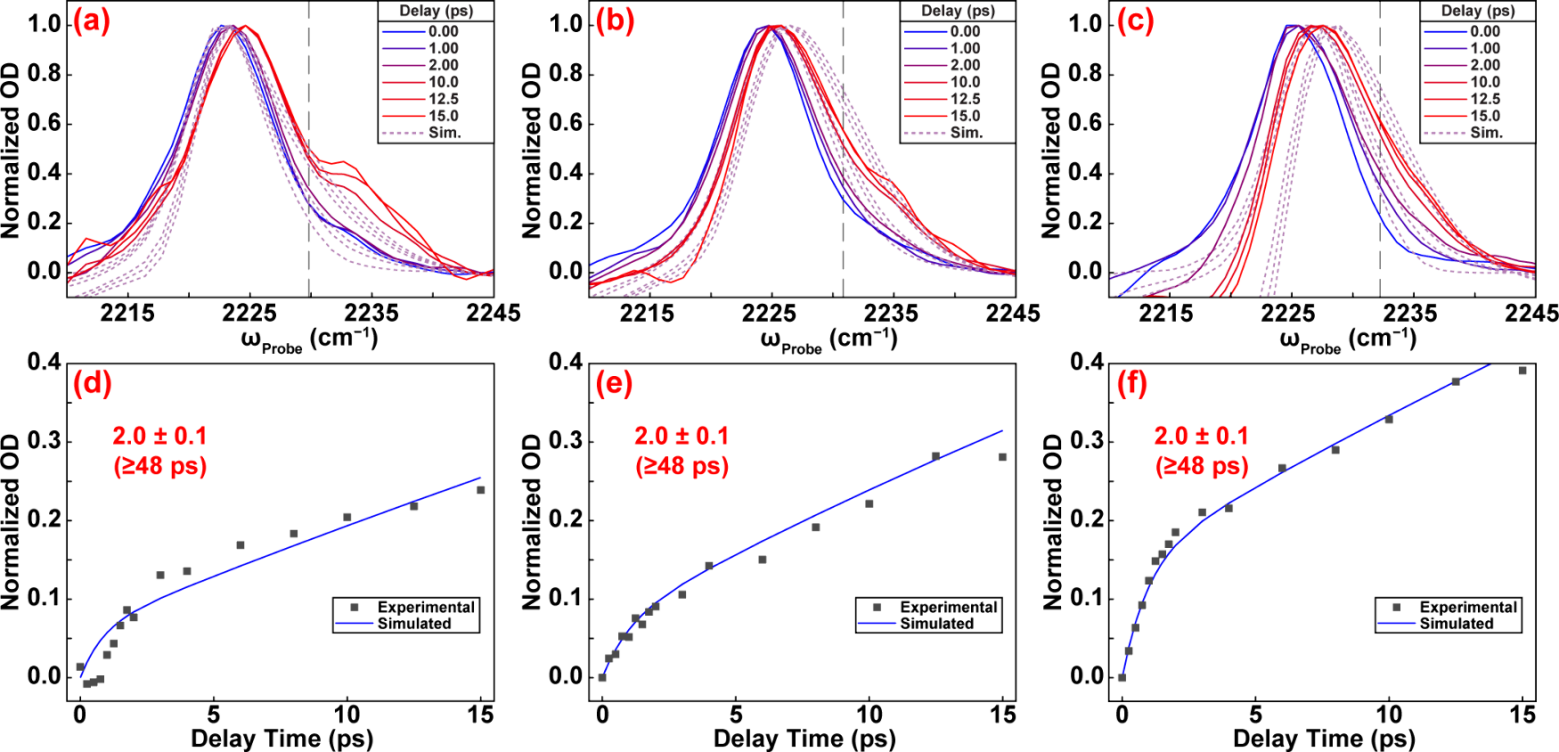
Comparison of MgCl_2_, LiCl, and KCl spectra and kinetics at +300 mV vs Ag/AgCl. Experimental pump slices (overlaid with simulated slices) from 15 ps spectra of 4-MBN in 100 mM (a) MgCl_2_, (b) LiCl, and (c) KCl. Kinetic traces of cross-peak evolution in experimental and simulated data for (d) MgCl_2_, (e) LiCl, and (f) KCl samples.

We performed MD simulations to assess ion distributions near the 4-MBN-modified surface and correlate them with measured chemical exchange rates. Simulation boxes were constructed following previous work, but instead of pure water, aqueous solutions of MgCl_2_, LiCl, and KCl were used.^31,63^ Sampling was accelerated using 0.5 M solutions; though higher than experiment, they remain dilute enough to avoid strong ion–ion correlations. Simulations were carried out at ±2 V using the constant charge method. A ±2 V potential was applied between W.E. and C.E. in MD simulations, which approximately corresponds to experimental W.E. vs R.E. potentials of −200 mV and +300 mV vs Ag/AgCl.^31^ Simulation and force field details are provided in Supporting Information. Shown in Figure 5 are radial distribution functions (RDF) for each ion at the two potentials as a function of its distance from the N atom of the 4-MBN cyano group. At –2 V, Mg^2+^ and Li^+^ accumulate at the interface modestly, while K^+^ has a markedly higher surface concentration. This difference likely arises from the lower hydration energy and larger radius of K^+^, which reduce its desolvation penalty near the interface. Mg^2+^ and Li^+^ are kosmotropic, maintaining long-lived hydration shells, making them less responsive to electrode potential. In contrast, K^+^, a known structure-breaker, accumulates more readily but disrupts interfacial water organization. At +2 V, the cations are repelled from the electrode, regardless of their identity, while Cl^–^ is enriched at the interface. The concentration of Cl^–^ at the interface is largely independent (±10%) of the corresponding cation, indicating that ion pairing plays a minor role compared to electrostatic attraction to the electrode.

**Figure 5:**
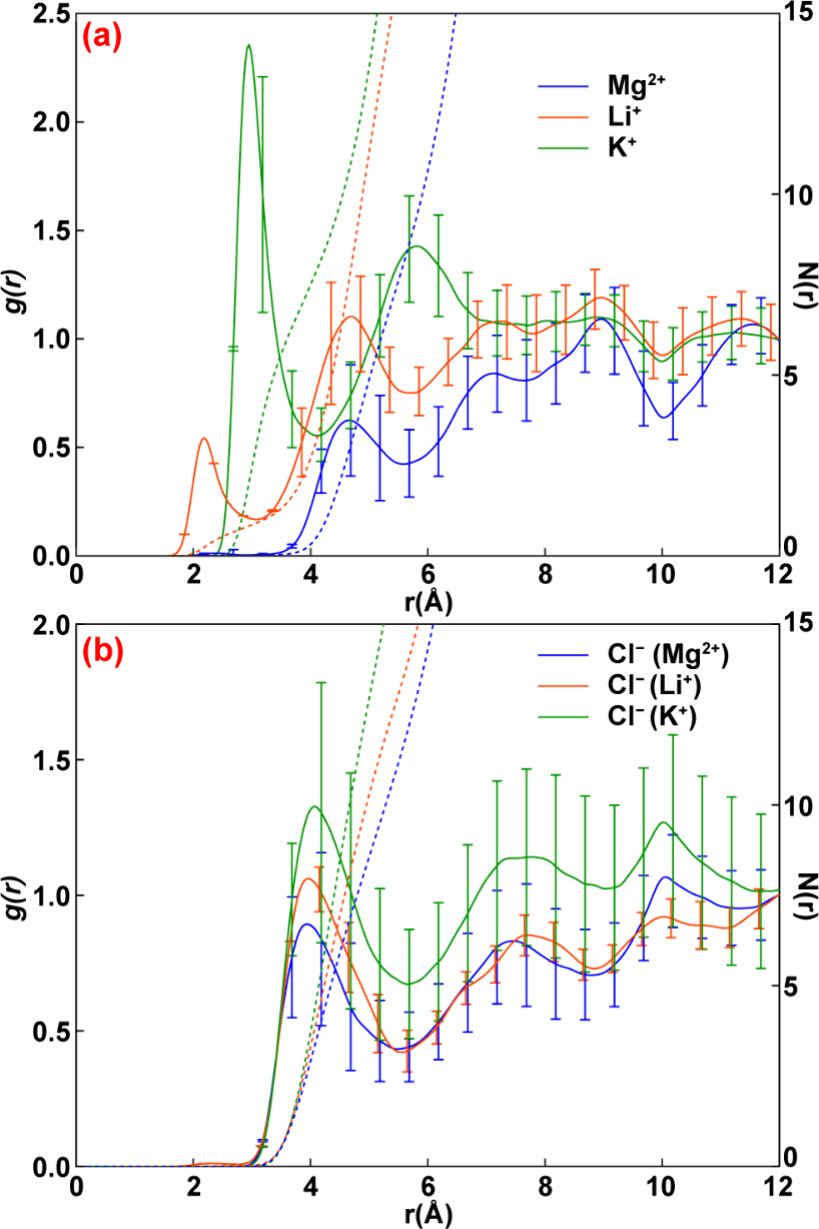
Radial distribution functions (solid) of ions around the N atom of the 4-MBN calculated from molecular dynamics simulations for (a) Mg^2+^, K^+^, Li^+^ at –2 V and (b) Cl^–^ at +2 V. Dotted lines are cumulative radial distribution functions.

We note that the chemical exchange rates correlate with interfacial ion densities predicted by simulation. At −200 mV, Mg^2+^ and Li^+^ exhibit moderate surface accumulation, while the experiments exhibit 34–40 ps (2.5–2.9 × 10^–2^ ps^–1^) exchange dynamics. In contrast, K^+^ accumulates more strongly, and the experiments have slower exchange kinetics. Thus, the correlation is consistent with the cation concentration at the surface hindering chemical exchange. At +300 mV, Cl^–^ concentrates at the interface, and the exchange kinetics are again slow. These results suggest that higher local ion densities, regardless of valency or sign, slow the chemical exchange kinetics between these two subpopulations of molecules. The solvation environment of the monolayer in terms of different types of 4-MBN molecules is summarized in Supporting Information Table S5. A noteworthy difference between the ions is the larger fraction of K^+^ ions that directly bind the nitrile group 1.35% vs 0.15% for Li^+^ ions and 0% for Mg^2+^. This difference may be sufficient to explain the slower dynamics of the monolayer in case of K^+^.

In our electrolyte-free study, exchange was assigned to voltage-dependent H-bonding dynamics between water and 4-MBN. Subsequent simulations by Kwac et al. revealed that these two subpopulations also differ in orientation (vertical at ∼ 13° and tilted at ∼ 36° from the surface normal).^57^ Because the calculated 4-MBN–water H-bond lifetimes are only 2–4 ps (Table S6), which is far shorter than the observed ≥33–48 ps kinetics, the measured exchange instead reflects reorientation of 4-MBN molecules within the monolayer, modulated by the electrolyte and applied potential. Exchange occurs on tens-of-picoseconds time scales, consistent with reorientation coupled with subpicoecond solvation changes similar to neat water.

At negative potential, the tens-of-ps chemical exchange differs for the three electrolytes, indicating ion-specific effects on monolayer dynamics. The fitted population ratios (Tables S3 and S4) show small but systematic electrolyte-dependent deviations in the equilibrium distribution between the two 4-MBN subensembles. These variations suggest that differences in interfacial solvation and hydrogen bonding subtly bias the relative stabilities of each orientational configuration. This is in stark contrast to the dynamics at a positive potential, where the chemical exchange rates are uniform. These differences in dynamics likely arise from electrolyte-dependent modulation of the energetic barrier between 4-MBN orientational configurations. Divalent and small monovalent ions (Mg^2+^, Li^+^) reduce the barrier between the two configurations, presumably by altering hydration structure since they bond with water strongly. Prior simulations at graphene interfaces found that applied electric fields can lower the energetic barrier between hydrogen-bonded and non-hydrogen-bonded configurations by reorganizing the local water structure.^64^ Kwac et al. find different solvation structures for the two 4-MBN geometries, and so the details of solvation structure may be important.^57^ Conversely, K^+^ and Cl^–^ suppress molecular dynamics, at the respective voltages that concentrate these ions at the surface, suggesting more direct interactions between salt and 4-MBN. Further simulations will disentangle these contributions. We note that it is unlikely that the electroinductive effect, which impacts the interpretation of Stark effect spectroscopy,^65,66^ is playing a role in the measured chemical exchange rates, because it is unlikely that the voltage-dependent electronic structure of 4-MBN depends on salt identity. The assignment of the two subpopulations to distinct orientational configurations is based on the combined experimental trends and MD simulations and should be viewed as the most consistent mechanistic interpretation of the data.

These 2D-IR experiments and simulations show electrode interfacial dynamics depend on voltage polarity and electrolyte. The voltage-dependent dynamics observed here are likely from the structural rearrangement of the 4-MBN molecules between two orientations. We deduce that the rate of exchange is impacted both by the identity of the salt through direct ion–molecule interactions and indirectly through altered solvent properties. The voltage-dependent dynamics of the EDL and that of molecules on the surface of electrodes are largely uncharacterized, despite their relevance to charge-screening, molecular recognition, and catalysis.^67–69^ Whether the Hofmeister series impacts dynamics as it does solvation remains to be studied. The experiments and simulations presented here offer a new approach to understanding how molecules and solvent respond to the voltage applied to a spectroelectrochemical cell.

## Supplementary Material



## Notes

Experimental methods, additional 2D-IR spectra, Kubo line shape simulation parameters, additional simulation and MD analysis, and supporting figures and tables (PDF)

## †

SkyWater Technology, Bloomington, MN 55425, USA; Email: mjryan97@gmail.com
