# Supplementary material for: Probing Voltage- and Electrolyte-Dependent Monolayer Dynamics with 2D-IR Spectroscopy
Source: J Am Chem Soc. 2025 Oct 24;147(44):40099–105. doi: 10.1021/jacs.5c14718 (PMC12593391; doi:10.1021/jacs.5c14718)
Supplement: Supplementary file 1 [file ja5c14718_si_001.pdf]

**Probing Voltage- and Electrolyte-Dependent Monolayer Dynamics with 2D-IR Spectroscopy**

*Austin B. Gilbert[1], Wonjae Jeong[1], Kyle R. Billings[2], Aritri Biswas[2], Matthew J. Ryan[1], Kijeong Kwac[3], Minhaeng Cho[3,4], Alexei A. Kananenka[2] and Martin T. Zanni[1]**

1.) Department of Chemistry, University of Wisconsin – Madison, Madison, Wisconsin 53706, USA

2.) Department of Physics and Astronomy, University of Delaware, Newark, Delaware 19716, USA

3.) Center for Molecular Spectroscopy and Dynamics, Institute for Basic Science (IBS), Seoul 02841, Republic of Korea

4.) Department of Chemistry, Korea University, Seoul 02841, Republic of Korea

# Supporting Information

## List of Supplementary Figures

**Figure S1.** 2D-IR spectra of 4-MBN in 100 mM $MgCl_2$ at −200 mV vs. Ag/AgCl, 0-15 ps delays ........ S5

**Figure S2.** 2D-IR spectra of 4-MBN in 100 mM $MgCl_2$ at +300 mV vs. Ag/AgCl, 0-15 ps delays ........ S5

**Figure S3.** 2D-IR spectra of 4-MBN in 100 mM LiCl at −200 mV vs. Ag/AgCl, 0-15 ps delays ........ S6

**Figure S4.** 2D-IR spectra of 4-MBN in 100 mM LiCl at +300 mV vs. Ag/AgCl, 0-15 ps delays ........ S6

**Figure S5.** 2D-IR spectra of 4-MBN in 100 mM KCl at −200 mV vs. Ag/AgCl, 0-15 ps delays ........ S7

**Figure S6.** 2D-IR spectra of 4-MBN in 100 mM KCl at +300 mV vs. Ag/AgCl, 0-15 ps delays ........ S7

**Figure S7.** $t_0$ diagonal of 4-MBN spectra at −200 mV vs. Ag/AgCl for $MgCl_2$, LiCl, and KCl ........ S8

**Figure S8.** $t_0$ diagonal of 4-MBN spectra at +300 mV vs. Ag/AgCl for $MgCl_2$, LiCl, and KCl ........ S8

**Figure S9-S20.** Experimental and simulated pump slices and kinetics at upper and lower bounds of the chemical exchange rate for each electrolyte and potential ........ S9-S14

**Figure S21.** Plane-averaged electrostatic potential profile vs. z-coordinate ........ S19

**Figure S22.** Average electric field profile along z-direction ........ S20

**Figure S23.** Water density distribution vs. z-coordinate ........ S21

**Figure S24.** Ion–N(4-MBN) radial distribution functions using full and ECC-scaled ion charges ........ S22

## List of Supplementary Tables

**Table S1.** Simulation parameters used in the two-population Kubo lineshape model fitted to −200 mV experimental data... S15

**Table S2.** Simulation parameters used in the two-population Kubo lineshape model fitted to +300 mV experimental data... S16

**Table S3.** The charges of the Au atoms used in constant charge simulations ........ S17

**Table S4.** Parameters of the force fields for the ions ........ S17

**Table S5.** Coordination environment of nitrile groups within 4-MBN monolayer ........ S17

**Table S6.** 4-MBN–water hydrogen-bond relaxation times for all electrolytes and potentials ........ S18

**Materials and Methods**

## *Electrochemical cell and sample preparation*

The fabrication and characterization of the electrochemical window and the spectroelectrochemical cell have been previously reported in detail.[3, 4] Calcium fluoride ($CaF_2$) electrode substrates were fabricated following the modified procedure as follows: 25 mm x 2 mm $CaF_2$ windows (Crystran) were descummed with downstream asher (YES CV200RFS, Wisconsin Centers for Nanoscale Technology - Nanoscale Fabrication Center (NFC)) for 2 min before deposition. A 7 nm indium tin oxide (ITO) was deposited via radio-frequency sputtering (Kurt J. Lesker PVD 70, 3 mTorr, 50 W). The ITO layer serves as an IR-transparent conductive layer, and the thickness and power settings are optimized for conductivity. A 1.5 nm aluminum oxide ($Al_2O_3$) layer was then sputtered (Telemark Dielectric Evaporator, NFC) on the ITO layer to provide buffer between ITO and gold, suppressing Fano lineshape distortion while maintaining optimal conductivity. On the edge of the windows, a 70 nm gold (Au) ring was deposited via physical vapor deposition (1.5 Å/s, Telemark Metal Evaporator, NFC) to enhance electrical contact. Above the $Al_2O_3$ buffer layer, a thin 3 nm Au layer was deposited via physical vapor deposition (0.1 Å/s, Telemark Metal Evaporator, NFC) for gold-thiolate monolayer tethering as well as plasmonic enhancement.

The spectroelectrochemical cell was assembled as previously described, using the corresponding electrolyte solution, in a static configuration with no pumping of electrolyte solution through the cell.[3, 4] Au electrodes were soaked overnight in 30 mM 4-MBN in ethanol, rinsed, and dried under nitrogen ($N_2$). A 3-electrode geometry was used, employing a plasmonic Au electrode (described above) as the working electrode (W.E.), a 3 mm platinum disc electrode (eDAQ), and a 2 mm leakless silver/silver chloride (Ag/AgCl) electrode (eDAQ) as the reference electrode (R.E.).

Electrolyte solutions, composed of potassium chloride (KCl), magnesium chloride hexahydrate ($MgCl_2{\cdot}6H_2O$), lithium chloride (LiCl) were all prepared at 100 mM concentrations in 18 mQ water. All electrolyte salts were used as-received and without further purification (ThermoFisher).

## *2D IR Spectroscopy*

The mid-infrared light for the 2D-IR experiments was generated using a Spectra-Physics Solstice 1kHz regenerative amplifier centered at 800 nm (4.4 W, 100 fs) to pump an optical parametric amplifier (Spectra-Physics TOPAS Prime). A silver gallium sulfide ($AgGaS_2$) difference frequency generation crystal is used to generate mid-IR light centered at 4.5 μm with ~100 fs long pulses. A CaF2 beamsplitter (90/10) is used to create the pump and probe lines. The pump beam is shaped using an acousto-optical modulator as previously described.[5] A 6.5 cm focal length parabolic mirror is used to overlap the pump and probe pulses spatially and temporally at the sample. The signal beam overlapped with the probe beam is directed into a monochromator (Princeton Instruments) with a 300 g/mm grating, which disperses the light onto a 64-pixel mercury cadmium telluride (MCT) array detector (Infrared Systems). The resolution of the probe is 1 $cm^{-1}$. All spectra are collected with a ZZXX polarization scheme, chosen to suppress pump–probe scatter; this geometry optimizes signal quality but is not intended to provide orientational selectivity. Polarization dependent 2D-IR measurements are unlikely to be informative, because the plasmonic gold surface does not support in-plane electric fields.[6]

## *Kubo Lineshape Simulations*

Lineshape simulations of experimental 2D-IR data were performed as described previously according to a two-population lineshape model.[3] This work adopted a two-population, three-correlation frequency-frequency correlation function (FFCF), as reported in literature to account for the presence of ions.[7] Parameters for the lineshape simulations of each dataset are shown in **Table S1-S2**. The simulated 2D-IR spectra are generated using a Kubo lineshape model containing

multiple FFCF parameters that together reproduce spectral diffusion at early delays (**Table S1-S2**). These parameters govern homogeneous and inhomogeneous broadening but do not directly correspond to measurable exchange rates. The only parameter that governs the long timescale growth of the cross-peak is the chemical exchange rate constant, which represents population transfer between the two subensembles. Accordingly, we report this rate (**Table 1**) as the physically meaningful timescale for comparing dynamics across electrolytes. Agreement between simulation and experiment was established by comparing both the pump slices and cross-peak kinetics. Specifically, the simulated pump slices reproduce the experimental lineshapes and intensities at each delay, while the simulated cross-peak kinetics follow the experimental cross-peak intensity evolution, supporting that the chosen parameters accurately capture the observed dynamics. Confidence intervals for reported chemical exchange rates were estimated by incrementally increasing and decreasing the exchange rate until the simulated pump slices, kinetic trend, or both no longer provided satisfactory agreement with the experimental data; the resulting upper and lower bounds for each electrolyte and potential, along with their corresponding simulations, are shown in Supporting Information (**Figure S9-S20**). At this bound, further decrease in $k_{ex}$ results in noticeable deviation between experimental and simulated lineshapes or kinetic trends, defining the confidence limit for this dataset.

## *Molecular Dynamics Simulations*

The system was built following previous reports.[3, 8] Initially, the simulation cell comprising two Au(111) electrodes was created using LAMMPS.[9] Each Au electrode was modeled using three sheets of Au atoms each containing 114 atoms. The two Au electrodes were separated by 73.0 Å. A monolayer of 4-mercaptobenzonitrile (4-MBN) molecules in a $(\sqrt{3}\ \times\ \sqrt{3})R30°$ formation was placed above one of the Au electrodes oriented with the sulfur atoms placed above the Au atoms. Absorption, and relaxation of 4-MBN was accomplished through a 200 ps gradual heating from 0 to 298 K under the canonical ensemble (NVT), and then keeping temperature constant at 298 K for 1 ns. We note that the average angle between the CN vector and the surface normal for 4-MBN monolayer we built is ~40º and the distribution of angles contains one peak. Compared to work by Kwak et. al. our monolayer is close to titled configuration and it is ordered.[10]

Separately, aqueous solutions of 0.5M of $MgCl_2$, LiCl, and KCl were created, minimized, and equilibrated for 600 ps under the constant number of particles, constant pressure, and constant temperature (NTP) conditions at 298K and 1 atm. Each solution included 1600 SPC/E water molecules.[11] The number of ions of $MgCl_2$ is 15 cations and 30 $Cl^-$ ions, whereas LiCl and KCl were made up of 15 of both cations and anions. The procedure was repeated 8 times, each time the location of ions in the box was chosen randomly and differently. We note that, while ion concentrations in MD simulations were higher than in the experiment, the solution remains sufficiently dilute that significant ion–ion interactions are not observed. Therefore, lower ion concentrations are not expected to noticeably affect the RDFs.

The equilibrated solution box is then combined with the electrode/4-MBN system. An 800 ps NPT simulation at 298 K and 1 atm was conducted to allow for changes in the z-direction while the x-direction and y-direction were held constant. The average final box size is 34.62 Å x 29.98 Å x 66.67 Å. An additional 1 ns NVT equilibration was performed to obtain the initial configurations for the constant charge simulations. The latter were performed for three 2V, 0V, and −2V by assigning charges of the innermost Au layer. For each voltage a 40 ns NVT equilibration was performed. The Poisson equation was then integrated based on the configurations from an equilibration trajectory to verify that the potential difference is the same as the desired voltage. The process was repeated, when necessary, to adjust the charges of the Au layer. The final charges for all three voltages are summarized in **Table S1**. We note that these charges agree with those used in constant potential simulations reported previously.[3, 8]

After an NVT equilibration at the set voltage, a 250 ps trajectory was generated under microcanonical (NVE) ensemble with the snapshots saved 10 fs and used for the analysis. All simulations were performed using LAMMPS with a 1 fs timestep.[9]

The force field parameters of 4-MBN are derived from the Amber GAFF force field, and the partial charges of 4-MBN were taken from Kwac et. al.[8, 12, 13] Previously defined Lennard Jones (LJ) parameters were used for the Au-Au interactions, and Morse potentials describing Au-S interaction potential were taken from Zhao et. al.[14] SPC/E force field was used for water. Ion force fields were taken from literature.[11] Ions were described within the electronic Continuum Correction (ECC) model.[15-19] ECC implicitly accounts for electronic polarization of water in ionic solutions. Within the ECC approach the ion charges are scaled by $1/\sqrt{\varepsilon_{el}} \approx 0.75$ to account for electronic polarization in water. $\varepsilon_{el} = 1.78$ is the dielectric permittivity of water. Rescaling of ion charges changes the balance between electrostatic and Lennard-Jones

(LJ) interactions so the LJ parameters are typically adjusted as well. **Table S4** contains all force field parameters for the ions. The long-range interactions were calculated by the particle–particle particle–mesh method, and all parameters between different atom types were determined using the Lorentz–Berthelot mixing rules.

We note that, in general electrode-electrolyte systems, an explicit treatment of molecular polarization using e.g., many-body potentials or *ab initio* molecular dynamics might be necessary.[20-22] In the system studied here the ECC treatment of polarization should be sufficient because water does not come near the electrode surface and the 4-MBN monolayer screens the ion-electrode interactions. **Figure S24** compares RDFs calculated using the ECC approach to those calculated using the full charges of the ions. The ECC approach leads to a much more structured distribution of ions, underscoring the importance of accounting for electronic polarization of water.

The electrostatic potentials and electric field profiles are shown in **Figure S21** and **S22**, respectively.

## 4-MBN-water hydrogen bonding

4-MBN-water hydrogen bonding is analyzed by defining the following time-correlation function:

$$S_{HB}(t) = \frac{\langle h(0)h(t)\rangle - \langle h\rangle^2}{\langle h\rangle}$$

where $h(t)$ is defined such that $h(t) = 1$ when a selected 4-MBN molecule in involved in H-bonding with any water molecule at time $t$ and $h(t) = 0$ otherwise and $\langle ... \rangle$ denotes the ensemble average. The hydrogen-bonding definition used here corresponds to N…H distance < 2.6 Å and O-H…N angle < 150°.

The obtained time-correlation function $S_{HB}(t)$ is fitted to a sum of three exponential functions $a_1 \exp\left(-\frac{t}{\tau_1}\right) + a_2 \exp\left(-\frac{t}{\tau_2}\right) + a_3 \exp\left(-\frac{t}{\tau_3}\right)$, with the condition $a_1 + a_2 + a_3 = 1$, to obtain the parameters $\tau_1, \tau_2, \tau_3$ shown in **Table S5**.

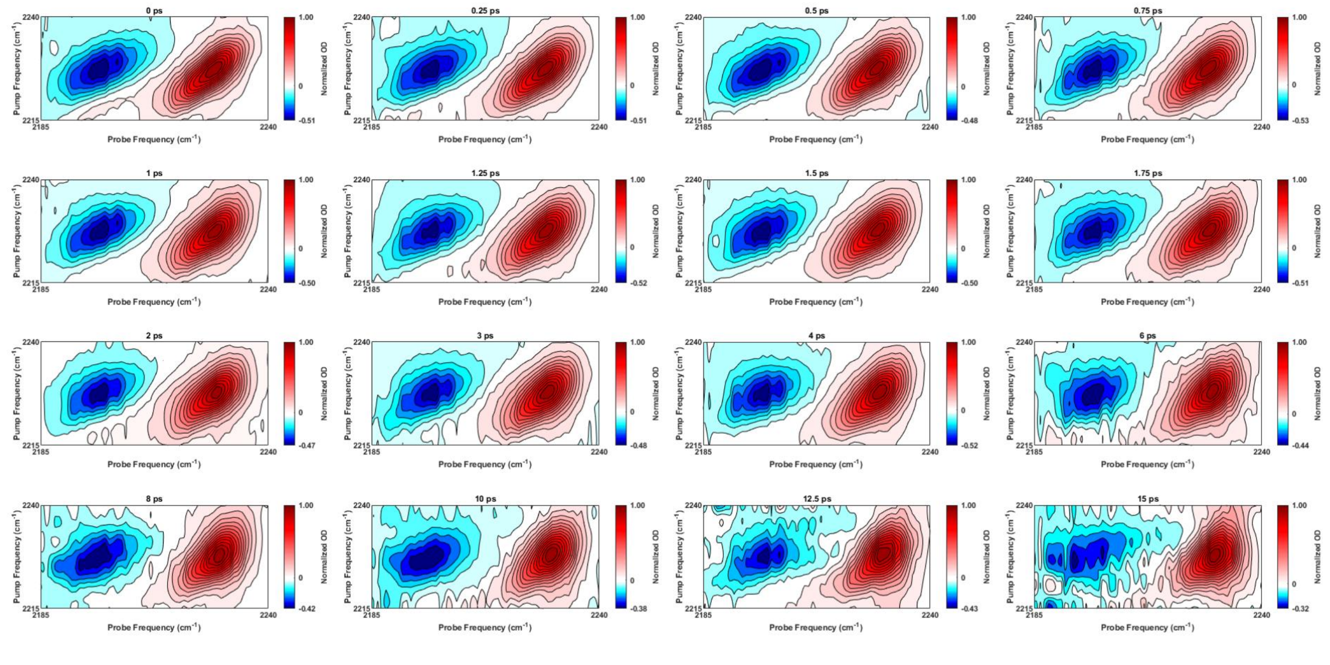


**Figure S1.** 2D-IR spectra of 4-MBN in 100 mM $MgCl_2$ at −200 mV vs. Ag/AgCl at delay times from 0-15 ps.

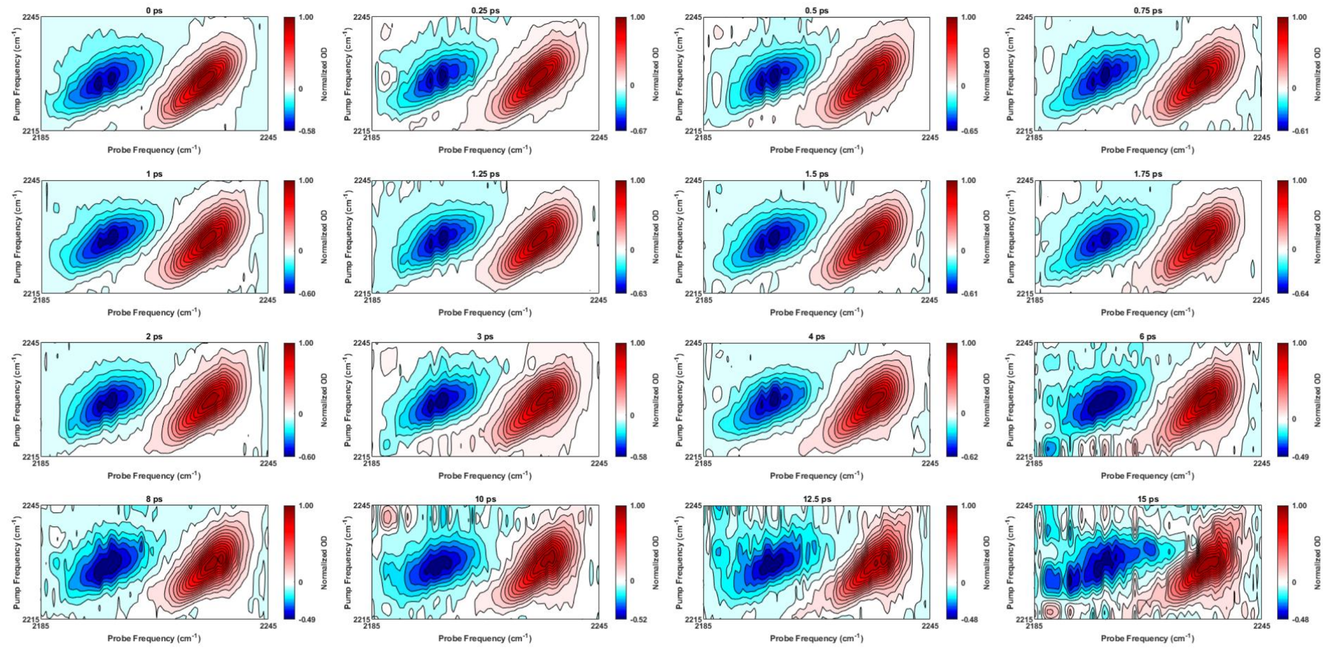


**Figure S2.** 2D-IR spectra of 4-MBN in 100 mM $MgCl_2$ at +300 mV vs. Ag/AgCl at delay times from 0-15 ps.

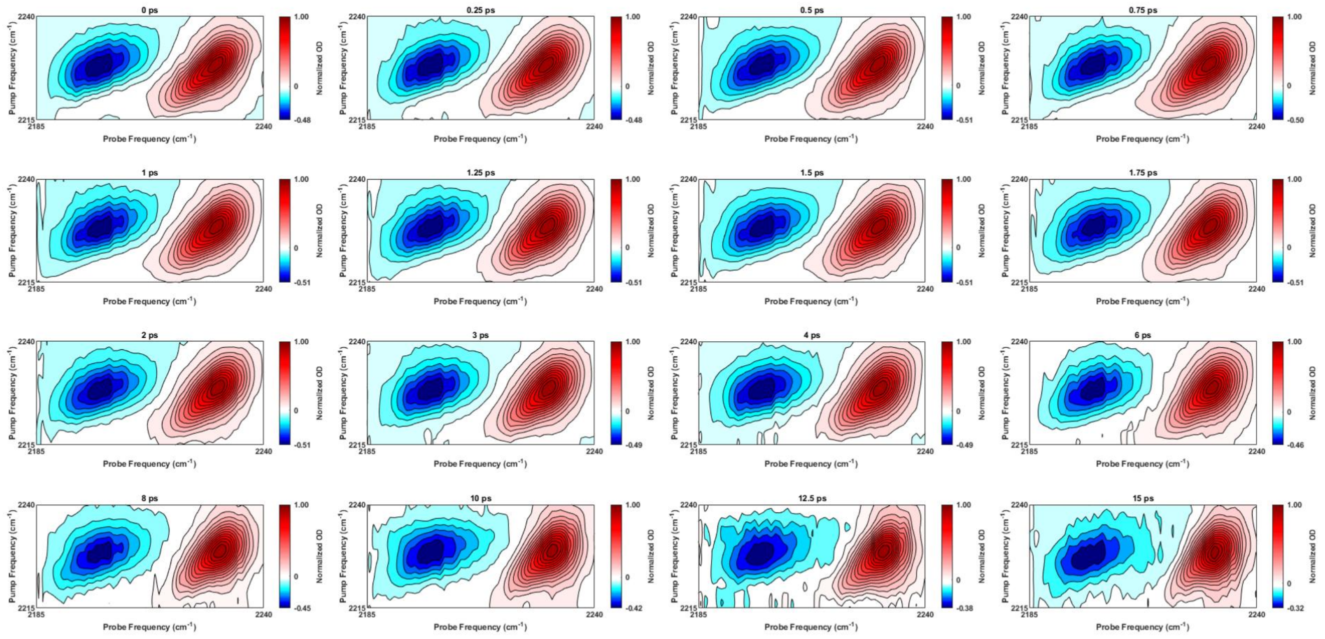

**Figure S3.** 2D-IR spectra of 4-MBN in 100 mM LiCl at −200 mV vs. Ag/AgCl at delay times from 0-15 ps.

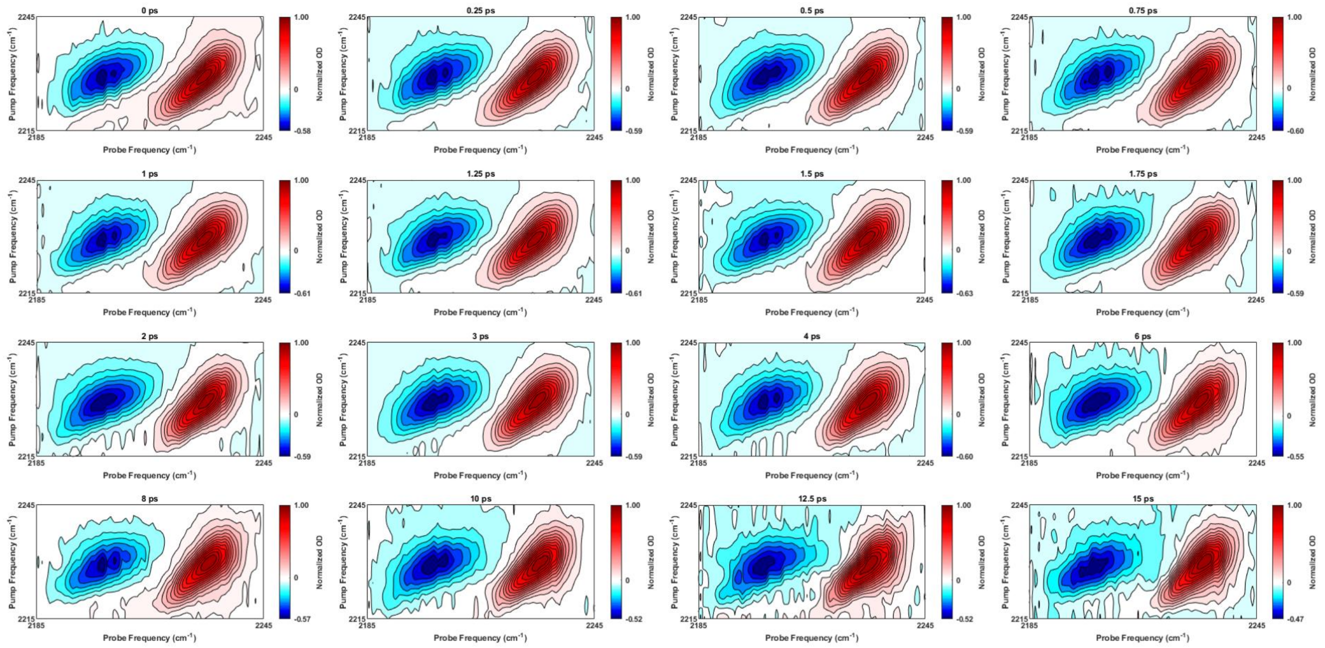

**Figure S4.** 2D-IR spectra of 4-MBN in 100 mM LiCl at +300 mV vs. Ag/AgCl at delay times from 0-15 ps.

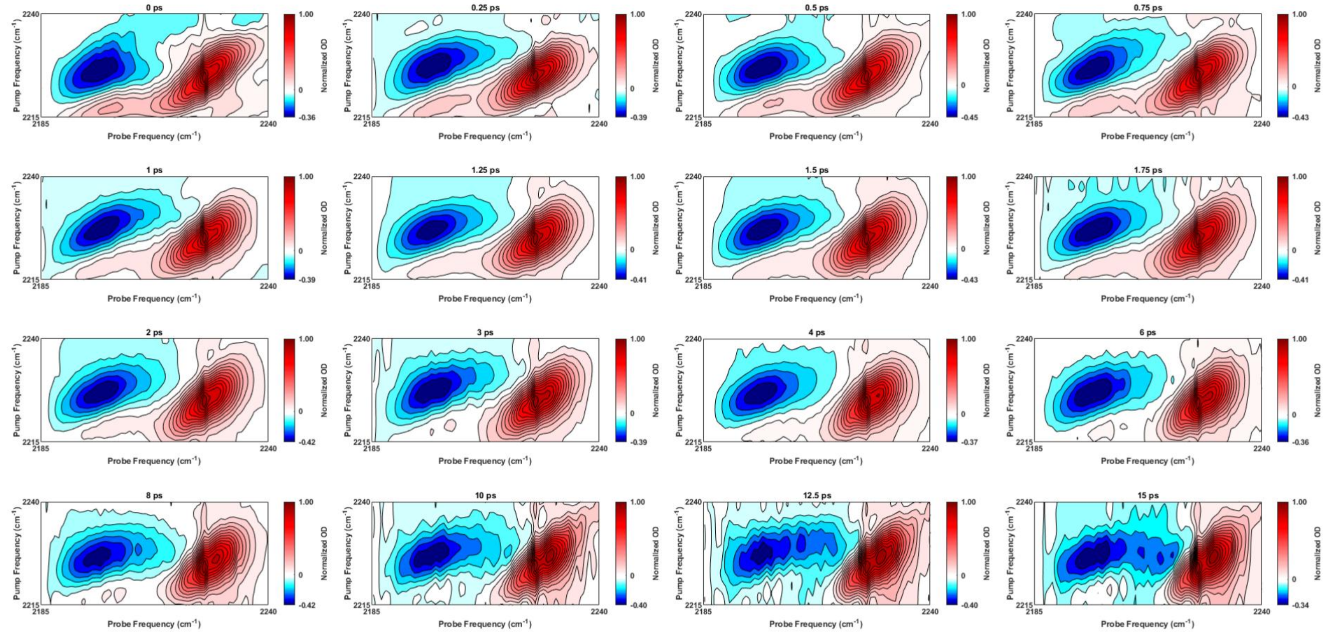


**Figure S5.** 2D-IR spectra of 4-MBN in 100 mM KCl at −200 mV vs. Ag/AgCl at delay times from 0-15 ps.

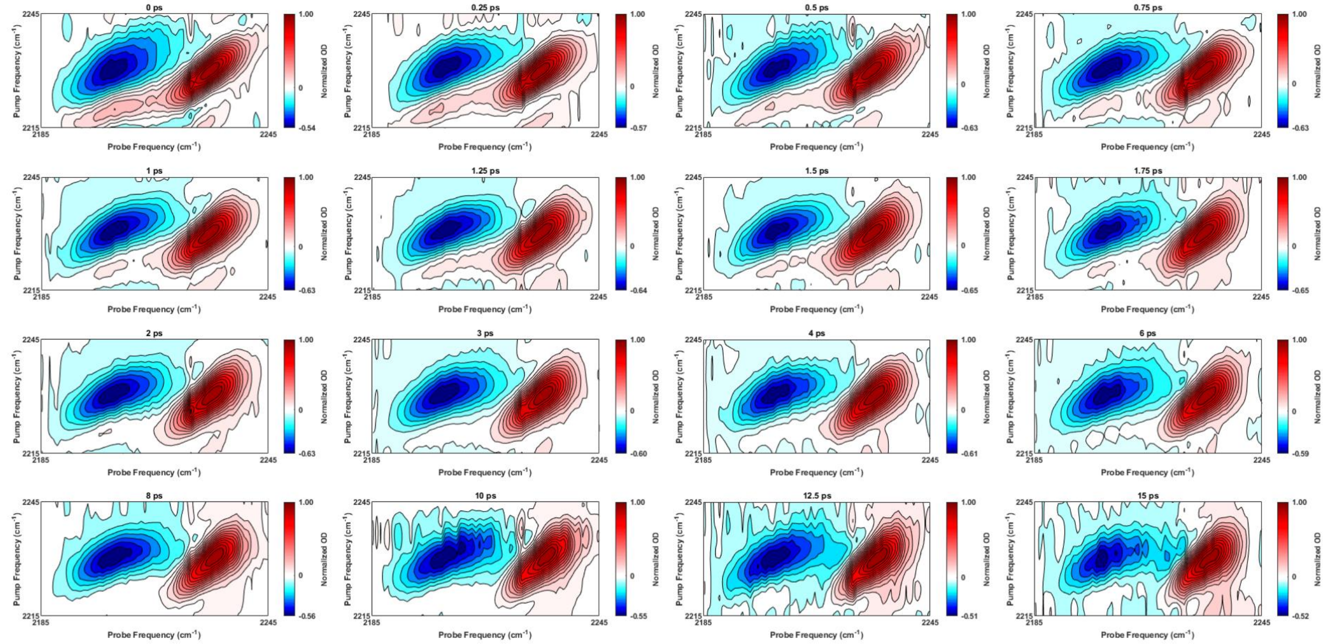


**Figure S6.** 2D-IR spectra of 4-MBN in 100 mM KCl at +300 mV vs. Ag/AgCl at delay times from 0-15 ps.

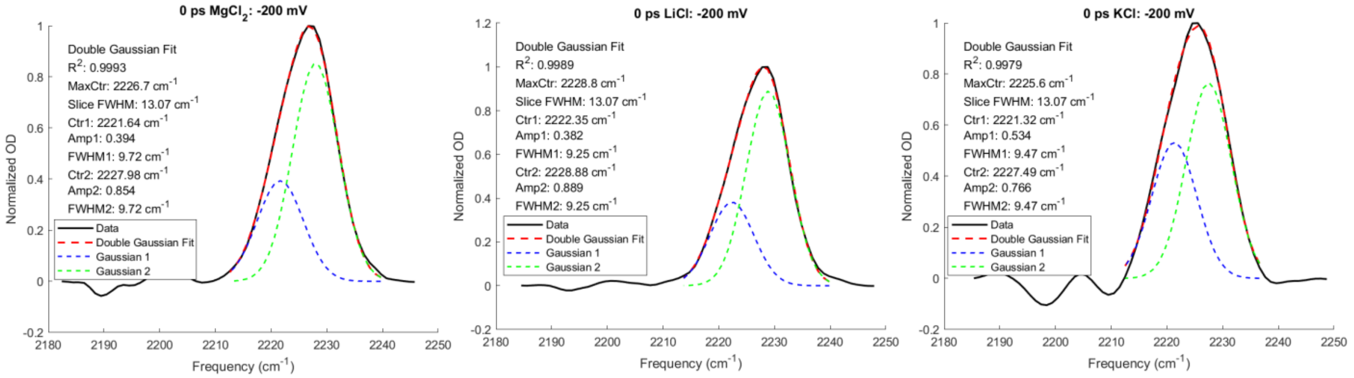


**Figure S7.** Diagonal cuts taken of $t_0$ spectra of 4-MBN at −200 mV vs. Ag/AgCl in 100 mM $MgCl_2$ (left), LiCl (middle), and KCl (right). Each slice is fit to the sum of two Gaussian populations.

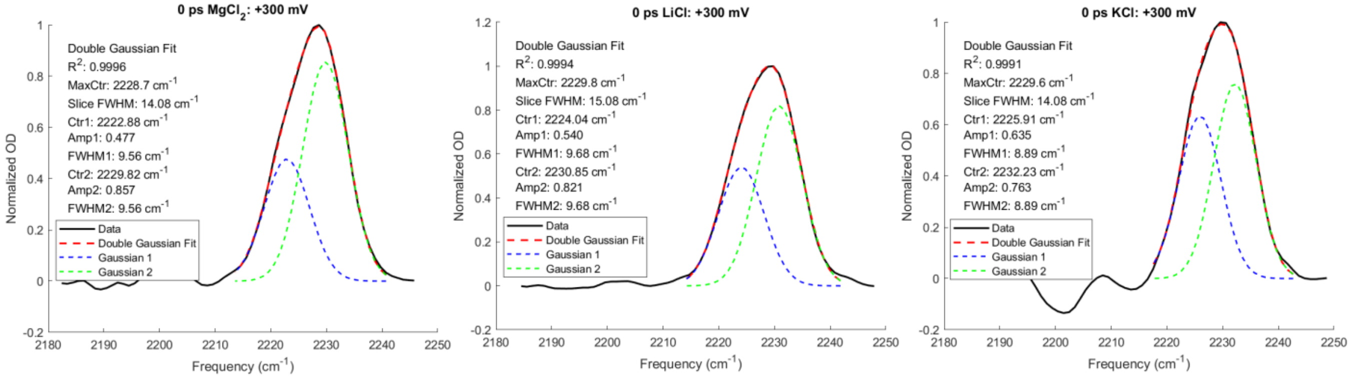


**Figure S8.** Diagonal cuts taken of $t_0$ spectra of 4-MBN at +300 mV vs. Ag/AgCl in 100 mM $MgCl_2$ (left), LiCl (middle), and KCl (right). Each slice is fit to the sum of two Gaussian populations.

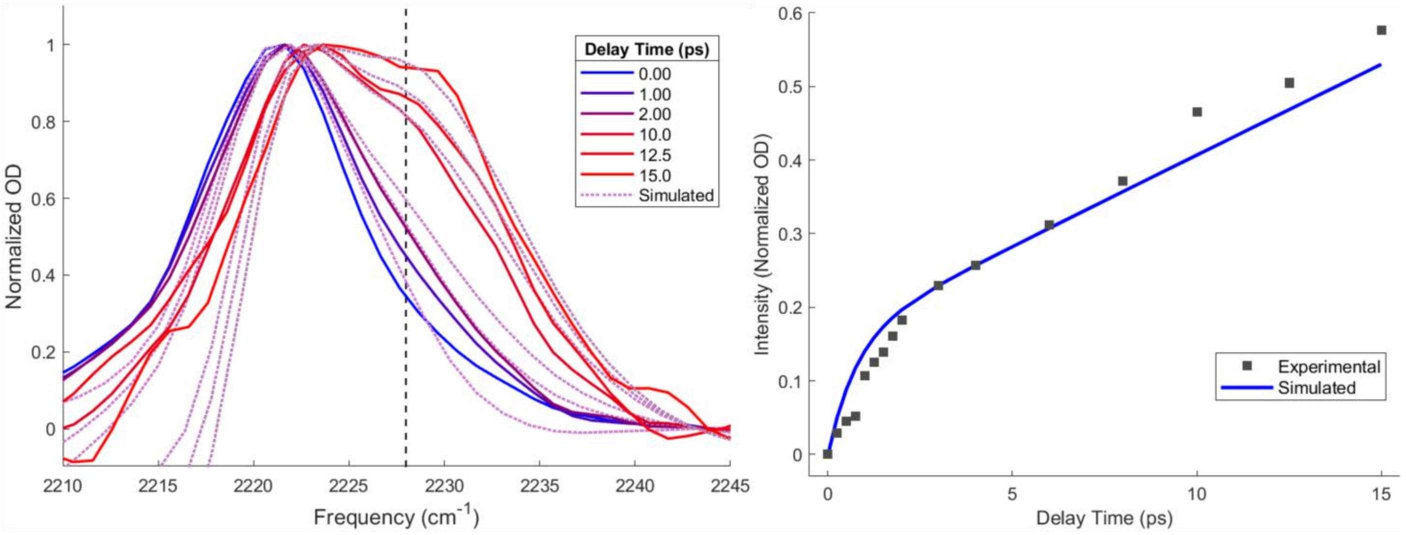


**Figure S9.** Experimental and simulated 2D-IR observables for $MgCl_2$ at −200 mV corresponding to the lower bound of the chemical exchange rate constant ($k_{ex}$) ($2.4 \times 10^{-2}$ $ps^{-1}$ (≥42 ps)). Experimental pump slices overlaid with simulated slices (left) and experimental cross-peak kinetics

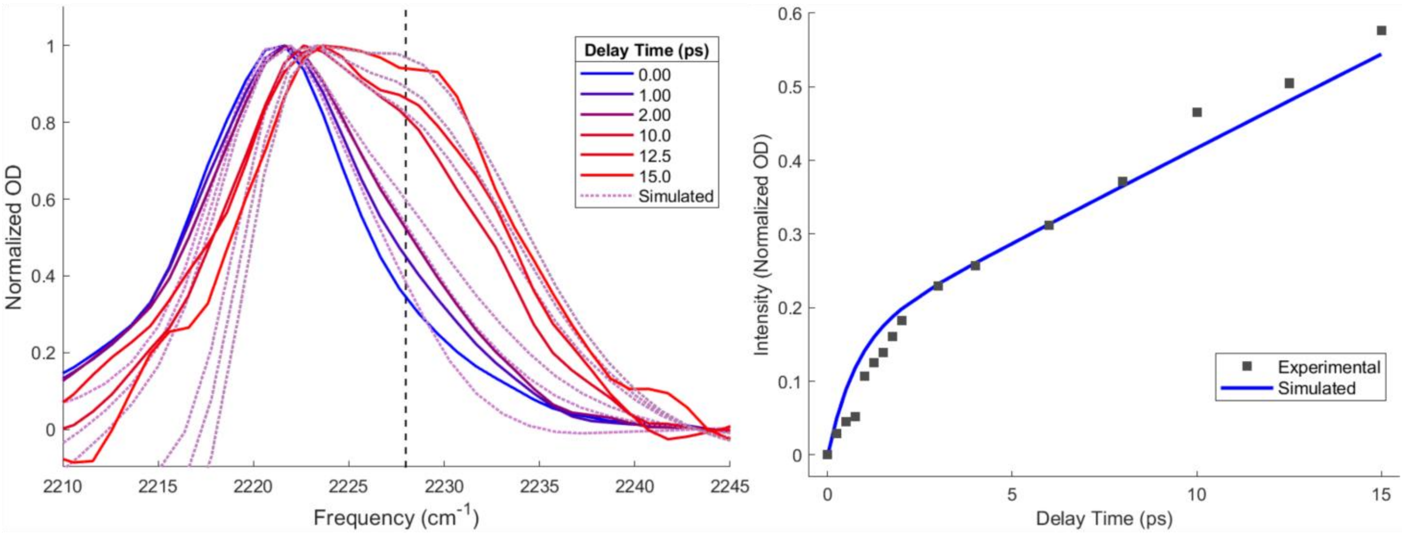


**Figure S10.** Experimental and simulated 2D-IR observables for $MgCl_2$ at −200 mV corresponding to the upper bound of the chemical exchange rate constant ($k_{ex}$) ($2.6 \times 10^{-2}$ $ps^{-1}$ (≥38 ps)). Experimental pump slices overlaid with simulated slices (left) and experimental cross-peak kinetics

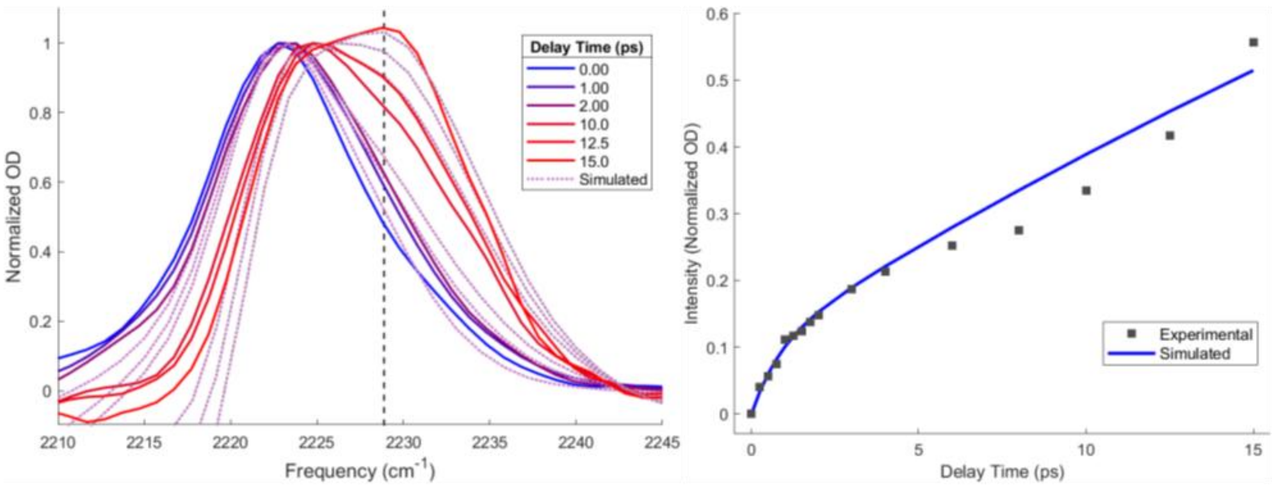


**Figure S11.** Experimental and simulated 2D-IR observables for LiCl at −200 mV corresponding to the lower bound of the chemical exchange rate constant ($k_{ex}$) ($2.8 \times 10^{-2}$ $ps^{-1}$ (≥36 ps)). Experimental pump slices overlaid with simulated slices (left) and experimental cross-peak kinetics overlaid with the simulated kinetic trend (right) are shown with the simulation using the indicated $k_{ex}$.

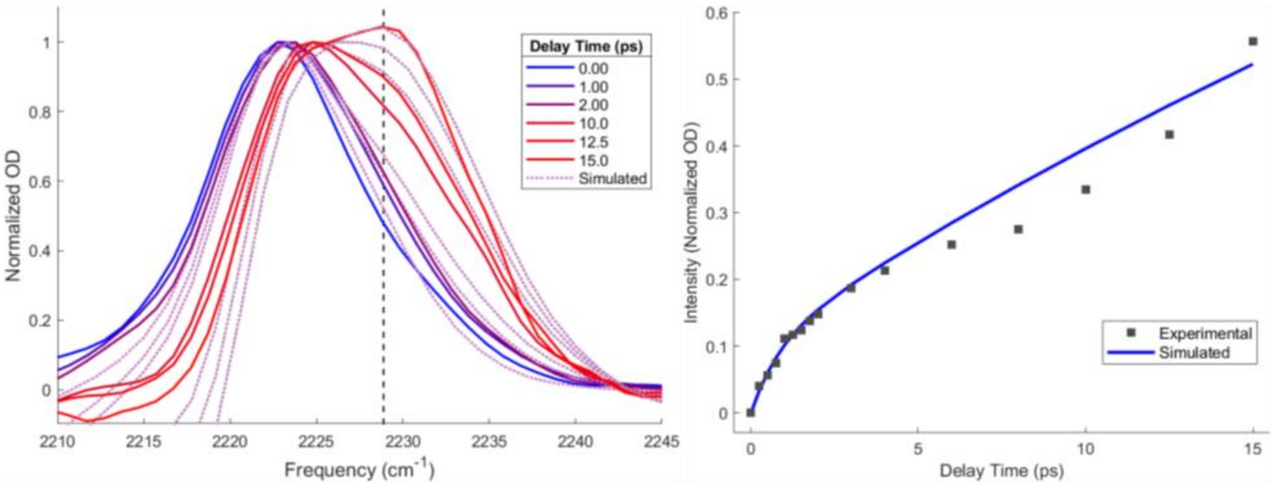


**Figure S12.** Experimental and simulated 2D-IR observables for LiCl at −200 mV corresponding to the upper bound of the chemical exchange rate constant ($k_{ex}$) ($3.0 \times 10^{-2}$ $ps^{-1}$ (≥33)). Experimental pump slices overlaid with simulated slices (left) and experimental cross-peak kinetics overlaid with the simulated kinetic trend (right) are shown with the simulation using the indicated $k_{ex}$.

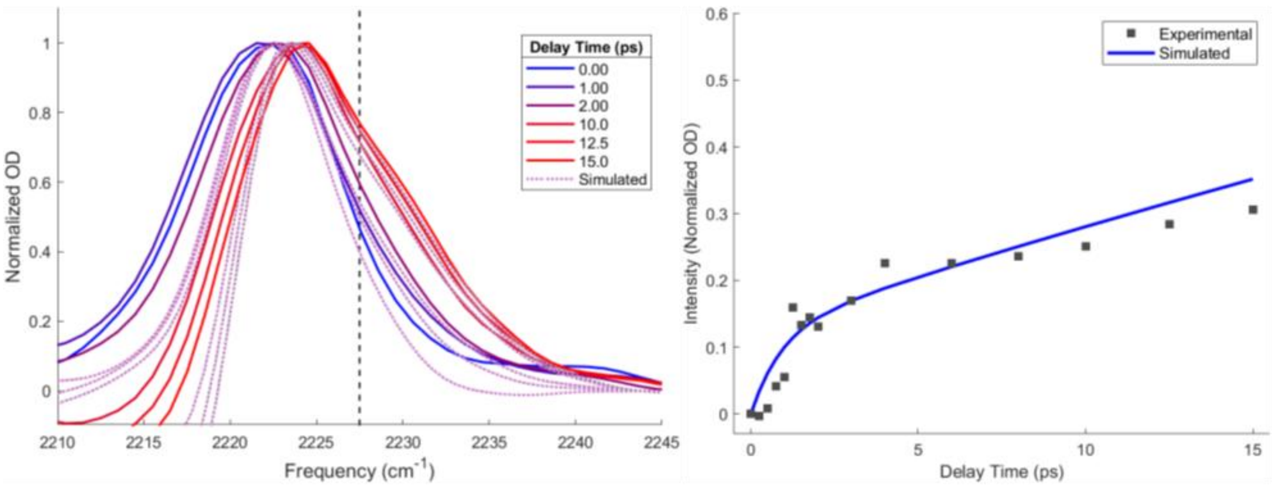


**Figure S13.** Experimental and simulated 2D-IR observables for KCl at −200 mV corresponding to the lower bound of the chemical exchange rate constant ($k_{ex}$) ($1.7 \times 10^{-2}$ $ps^{-1}$ (≥59 ps)). Experimental pump slices overlaid with simulated slices (left) and experimental cross-peak kinetics overlaid with the simulated kinetic trend (right) are shown with the simulation using the indicated $k_{ex}$.

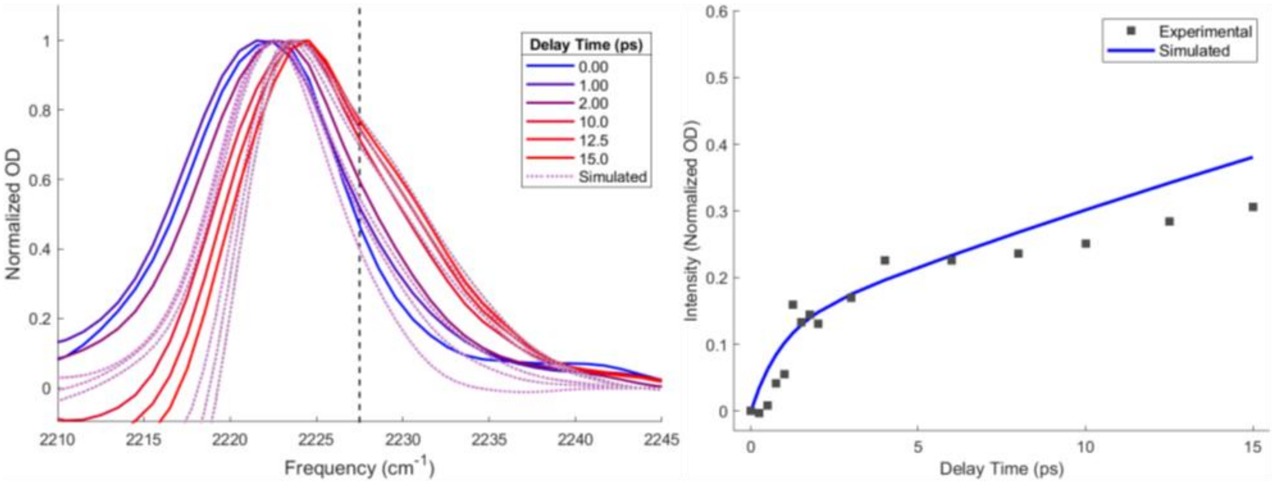


**Figure S14.** Experimental and simulated 2D-IR observables for KCl at −200 mV corresponding to the upper bound of the chemical exchange rate constant ($k_{ex}$) ($2.3 \times 10^{-2}$ $ps^{-1}$ (≥43 ps)). Experimental pump slices overlaid with simulated slices (left) and experimental cross-peak kinetics overlaid with the simulated kinetic trend (right) are shown with the simulation using the indicated $k_{ex}$.

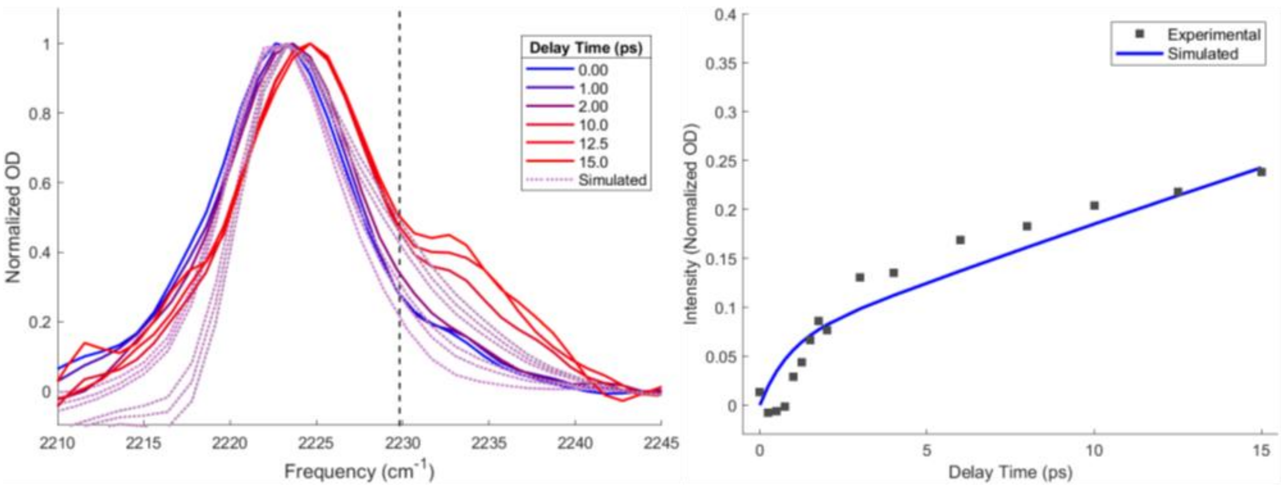


**Figure S15.** Experimental and simulated 2D-IR observables for $MgCl_2$ at +300 mV corresponding to the lower bound of the chemical exchange rate constant ($k_{ex}$) ($1.9 \times 10^{-2}$ ps$^{-1}$ (≥53 ps)). Experimental pump slices overlaid with simulated slices (left) and experimental cross-peak kinetics overlaid with the simulated kinetic trend (right) are shown with the simulation using the indicated $k_{ex}$.

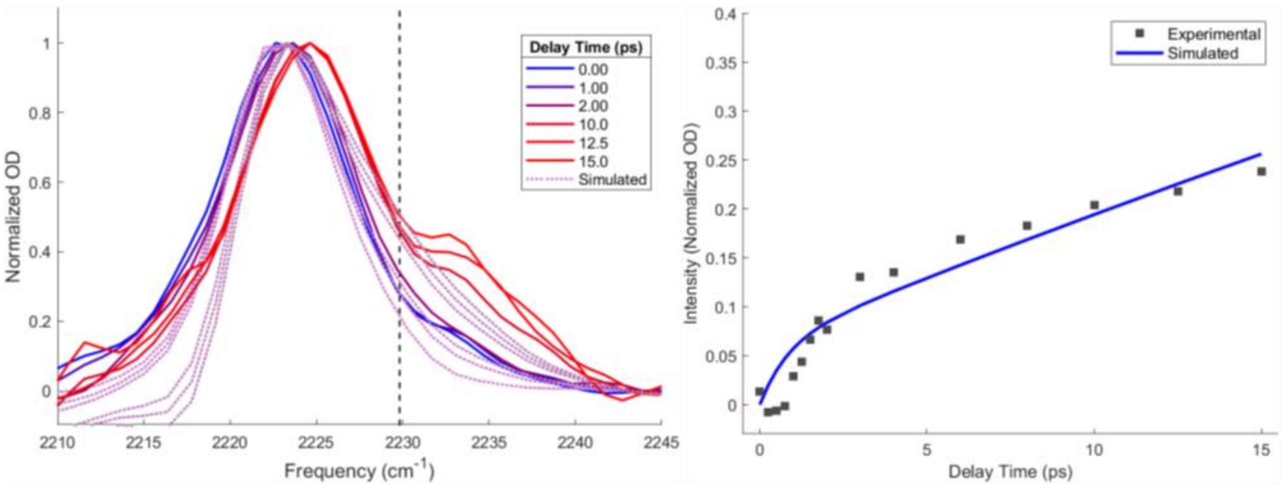


**Figure S16.** Experimental and simulated 2D-IR observables for $MgCl_2$ at +300 mV corresponding to the upper bound of the chemical exchange rate constant ($k_{ex}$) ($2.1 \times 10^{-2}$ ps$^{-1}$ (≥48 ps)). Experimental pump slices overlaid with simulated slices (left) and experimental cross-peak kinetics overlaid with the simulated kinetic trend (right) are shown with the simulation using the indicated $k_{ex}$.

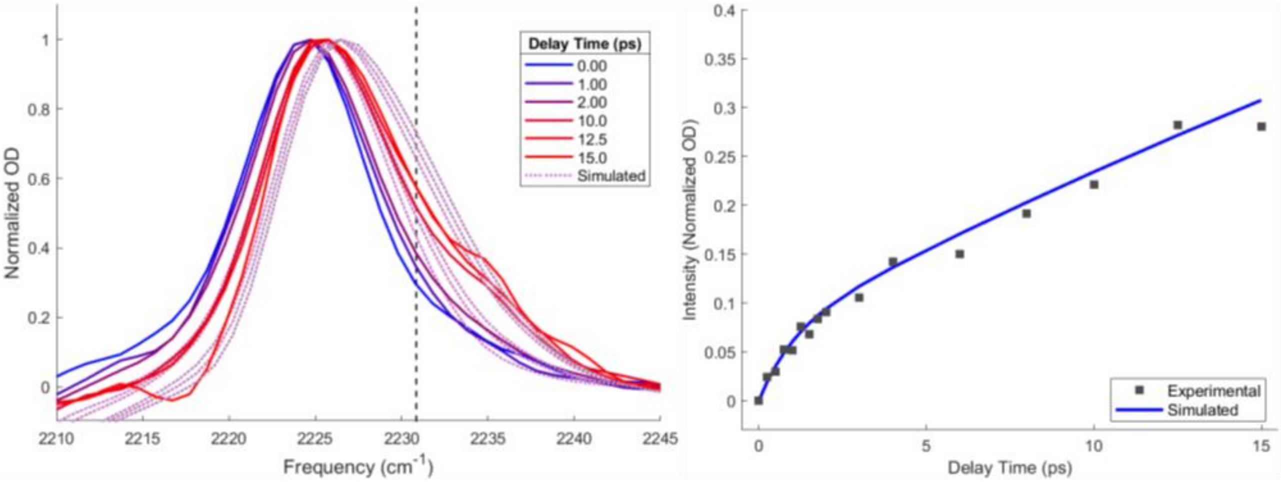


**Figure S17.** Experimental and simulated 2D-IR observables for LiCl at +300 mV corresponding to the lower bound of the chemical exchange rate constant ($k_{ex}$) ($1.9 \times 10^{-2}$ ps$^{-1}$ (≥53 ps)). Experimental pump slices overlaid with simulated slices (left) and experimental cross-peak kinetics overlaid with the simulated kinetic trend (right) are shown with the simulation using the indicated $k_{ex}$.

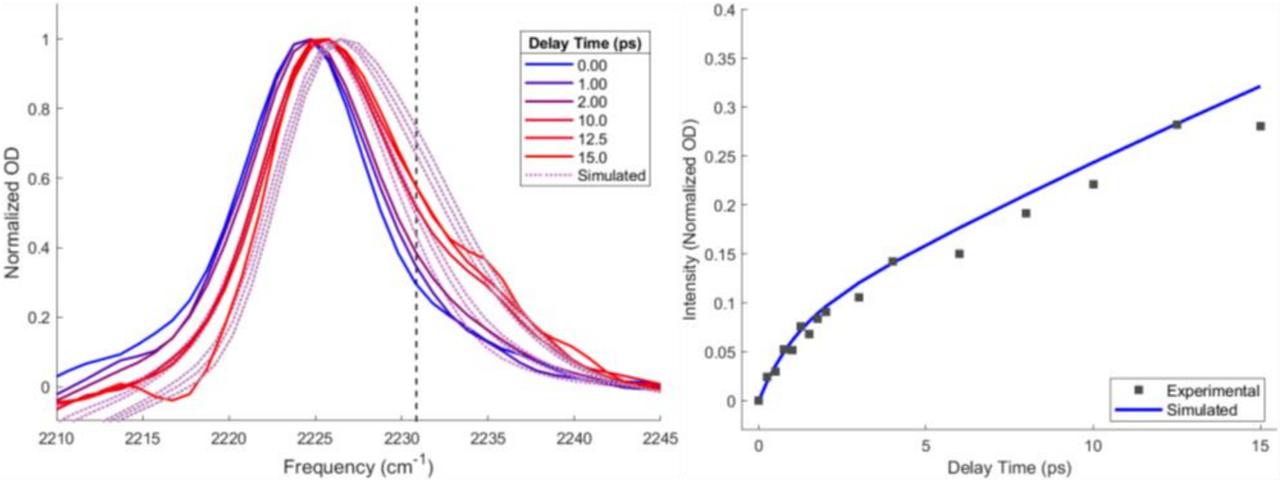


**Figure S18.** Experimental and simulated 2D-IR observables for LiCl at +300 mV corresponding to the upper bound of the chemical exchange rate constant ($k_{ex}$) ($2.1 \times 10^{-2}$ ps$^{-1}$ (≥48 ps)). Experimental pump slices overlaid with simulated slices (left) and experimental cross-peak kinetics overlaid with the simulated kinetic trend (right) are shown with the simulation using the indicated $k_{ex}$.

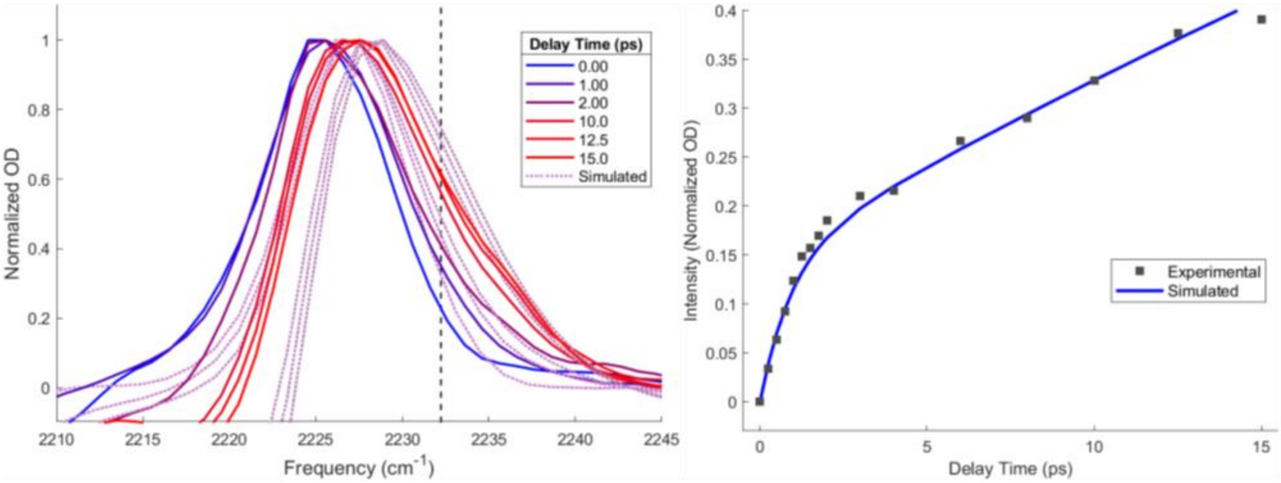


**Figure S19.** Experimental and simulated 2D-IR observables for KCl at +300 mV corresponding to the lower bound of the chemical exchange rate constant ($k_{ex}$) ($1.9 \times 10^{-2}$ ps$^{-1}$ (≥53 ps)). Experimental pump slices overlaid with simulated slices (left) and experimental cross-peak kinetics overlaid with the simulated kinetic trend (right) are shown with the simulation using the indicated $k_{ex}$.

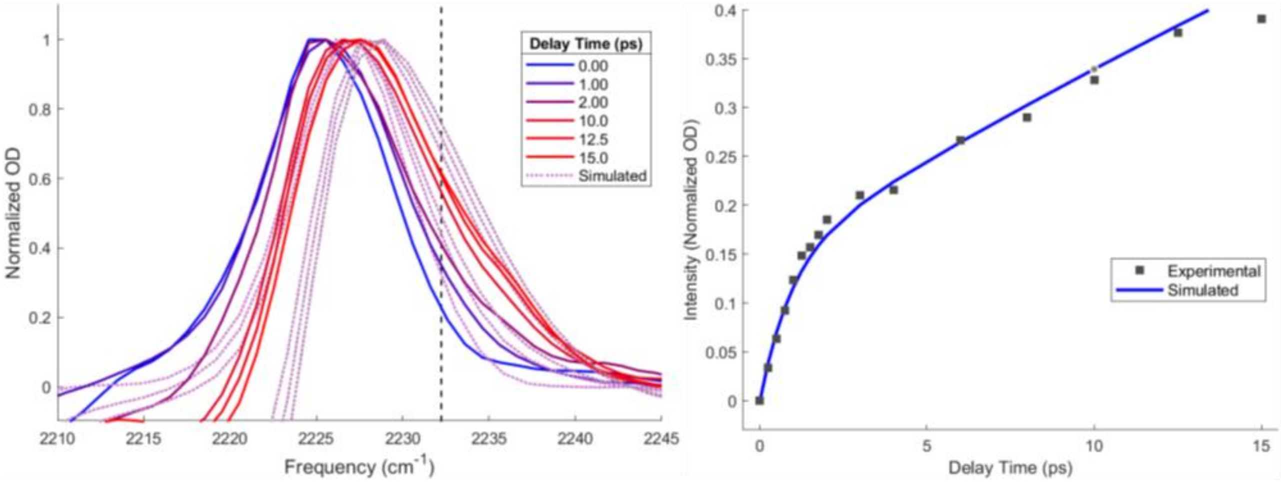


**Figure S20.** Experimental and simulated 2D-IR observables for KCl at +300 mV corresponding to the upper bound of the chemical exchange rate constant ($k_{ex}$) ($2.1 \times 10^{-2}$ ps$^{-1}$ (≥48 ps)). Experimental pump slices overlaid with simulated slices (left) and experimental cross-peak kinetics overlaid with the simulated kinetic trend (right) are shown with the simulation using the indicated $k_{ex}$.

**Table S1**. Center frequencies, correlation times, chemical exchange rates, and other parameters used in the two-population Kubo lineshape model fitted to −200 mV experimental data.

| −200 mV (vs. Ag/AgCl) | $MgCl_2$ | LiCl | KCl |
|---|---|---|---|
| $\omega_{CN(A)}$ ($cm^{-1}$) | 2222 | 2222 | 2221 |
| CN(A) Anharmonicity ($cm^{-1}$) | 30 | 30 | 30 |
| $\omega_{CN(B)}$ ($cm^{-1}$) | 2228 | 2229 | 2227 |
| CN(B) Anharmonicity ($cm^{-1}$) | 27 | 27 | 27 |
| $\Delta\omega_{A1}$ ($cm^{-1}$) | 1.00 | 1.00 | 1.00 |
| $\tau_{c(A1)}$ (ps) | 0.15 | 0.15 | 0.15 |
| $\Delta\omega_{B1}$ ($cm^{-1}$) | 1.50 | 1.50 | 1.50 |
| $\tau_{c(B1)}$ (ps) | 0.15 | 0.15 | 0.15 |
| $\Delta\omega_{A2}$ ($cm^{-1}$) | 4.50 | 4.50 | 4.00 |
| $\tau_{c(A2)}$ (ps) | 0.62 | 0.62 | 0.75 |
| $\Delta\omega_{B2}$ ($cm^{-1}$) | 6.50 | 6.20 | 5.50 |
| $\tau_{c(B2)}$ (ps) | 1.20 | 1.20 | 1.50 |
| $\Delta\omega_{A3}$ ($cm^{-1}$) | 5.00 | 5.00 | 4.50 |
| $\tau_{c(A3)}$ (ps) | $1.0 \times 10^6$ | $1.0 \times 10^6$ | $1.0 \times 10^6$ |
| $\Delta\omega_{B3}$ ($cm^{-1}$) | 2.50 | 2.50 | 3.50 |
| $\tau_{c(B3)}$ (ps) | $1.0 \times 10^6$ | $1.0 \times 10^6$ | $1.0 \times 10^6$ |
| Chemical Exchange Rate ($k_{ex}$, $ps^{-1}$) (Exchange time (ps)) | $2.5\ (\pm 0.1) \times 10^{-2}$ (≥38) | $2.9\ (\pm 0.1) \times 10^{-2}$ (≥33) | $2.0\ (\pm 0.3) \times 10^{-2}$ (≥43) |
| Vibrational Lifetime (ps) | 10.0 | 10.0 | 10.0 |
| IVR Time ($T_{IVR}$, ps) | 15.0 | 15.0 | 15.0 |
| Population Ratio ($n_A$:$n_b$) | 0.32:0.68 | 0.30:0.70 | 0.41:0.59 |

**Table S2**. Center frequencies, correlation times, chemical exchange rates, and other parameters used in the two-population Kubo lineshape model fitted to +300 mV experimental data.

| **+300 mV (vs. Ag/AgCl)** | **$MgCl_2$** | **LiCl** | **KCl** |
|---|---|---|---|
| **$\omega_{CN(A)}$ ($cm^{-1}$)** | 2223 | 2224 | 2226 |
| **CN(A) Anharmonicity ($cm^{-1}$)** | 30 | 30 | 30 |
| **$\omega_{CN(B)}$ ($cm^{-1}$)** | 2230 | 2231 | 2232 |
| **CN(B) Anharmonicity ($cm^{-1}$)** | 27 | 27 | 27 |
| **$\Delta\omega_{A1}$ ($cm^{-1}$)** | 1.00 | 1.00 | 1.00 |
| **$\tau_{c(A1)}$ (ps)** | 0.15 | 0.15 | 0.15 |
| **$\Delta\omega_{B1}$ ($cm^{-1}$)** | 1.50 | 1.50 | 1.50 |
| **$\tau_{c(B1)}$ (ps)** | 0.15 | 0.15 | 0.15 |
| **$\Delta\omega_{A2}$ ($cm^{-1}$)** | 4.50 | 4.50 | 4.50 |
| **$\tau_{c(A2)}$ (ps)** | 0.62 | 0.62 | 0.62 |
| **$\Delta\omega_{B2}$ ($cm^{-1}$)** | 4.50 | 4.50 | 4.50 |
| **$\tau_{c(B2)}$ (ps)** | 1.20 | 1.20 | 1.20 |
| **$\Delta\omega_{A3}$ ($cm^{-1}$)** | 3.00 | 5.00 | 5.00 |
| **$\tau_{c(A3)}$ (ps)** | $1.0 \times 10^6$ | $1.0 \times 10^6$ | $1.0 \times 10^6$ |
| **$\Delta\omega_{B3}$ ($cm^{-1}$)** | 3.50 | 3.0 | 3.00 |
| **$\tau_{c(B3)}$ (ps)** | $1.0 \times 10^6$ | $1.0 \times 10^6$ | $1.0 \times 10^6$ |
| **Chemical Exchange Rate ($k_{ex}$, $ps^{-1}$) (Exchange time (ps))** | 2.0 (± 0.1) $\times 10^{-2}$ (≥48) | 2.0 (± 0.1) $\times 10^{-2}$ (≥48) | 2.0 (± 0.1) $\times 10^{-2}$ (≥48) |
| **Vibrational Lifetime (ps)** | 10.0 | 10.0 | 10.0 |
| **IVR Time ($T_{IVR}$, ps)** | 35.0 | 35.0 | 15.0 |
| **Population Ratio ($n_A$:$n_b$)** | 0.36:0.64 | 0.40:0.60 | 0.45:0.55 |

**Table S3.** The charges of the Au atoms used in constant charge simulations.

| Target Voltage | 4-MBN electrode charge | Bare electrode charge |
|---|---|---|
| +2 V | −0.0035 e | +0.0035 e |
| 0 V | −0.0120 e | +0.0120 e |
| −2 V | −0.0211 e | +0.0211 e |

**Table S4.** Parameters of the force fields for the ions.

| | Ions | Charge | $\epsilon_i$ (kcal/mol) | $\sigma_i$ (Å) |
|---|---|---|---|---|
| $MgCl_2$[1] | $Mg^{2+}$ | +1.50 e | 0.8750 | 1.360 |
| | $Cl^-$ | −0.75 e | 0.1178 | 4.100 |
| LiCl[2] | $Li^+$ | +0.75 e | 0.0183 | 1.800 |
| | $Cl^-$ | −0.75 e | 0.1178 | 4.100 |
| KCl[2] | $K^+$ | +0.75 e | 0.1000 | 3.154 |
| | $Cl^-$ | −0.75 e | 0.1178 | 4.100 |

**Table S5.** The coordination environment of the 4-MBN monolayer. Percentage of the nitrile groups that belong to four types. Type 1 corresponds to the free nitrile groups. Type 2 are the nitrile groups which are hydrogen bonded to a water molecule which is found in the first hydration shell of an ion. Type 3 are the cation-bound nitrile groups. Type 4 are the nitrile groups hydrogen bonded to a water molecule which is not a part of the first hydration shell of an ion.

| Ion | Potential [V] | Type 1 (%) | Type 2 (%) | Type 3 (%) | Type 4 (%) |
|---|---|---|---|---|---|
| $K^+$ | -2 | 62.5 | 0.48 | 1.35 | 36.0 |
| $Li^+$ | -2 | 65.9 | 0.15 | 0.34 | 33.6 |
| $Mg^{2+}$ | -2 | 68.3 | 0.00 | 0.01 | 31.7 |
| $Cl^-$ (KCl) | +2 | 71.4 | - | 0.00 | 28.7 |
| $Cl^-$ (LiCl) | +2 | 68.0 | - | 0.00 | 32.0 |
| $Cl^-$ ($MgCl_2$) | +2 | 72.5 | - | 0.00 | 27.5 |

**Table S6.** The relaxation time constants (correlation times) of 4-MBN-water hydrogen bonding for all electrolytes and potentials studied in this work.

| System | Potential [V] | $\tau_1$ [ps] | $\tau_2$ [ps] | $\tau_3$ [ps] |
|---|---|---|---|---|
| LiCl | 2 | 0.012 | 0.17 | 2.39 |
| | −2 | 0.013 | 0.18 | 2.61 |
| KCl | 2 | 0.012 | 0.22 | 2.72 |
| | −2 | 0.014 | 0.35 | 3.68 |
| $MgCl_2$ | 2 | 0.012 | 0.15 | 2.50 |
| | −2 | 0.012 | 0.17 | 2.70 |

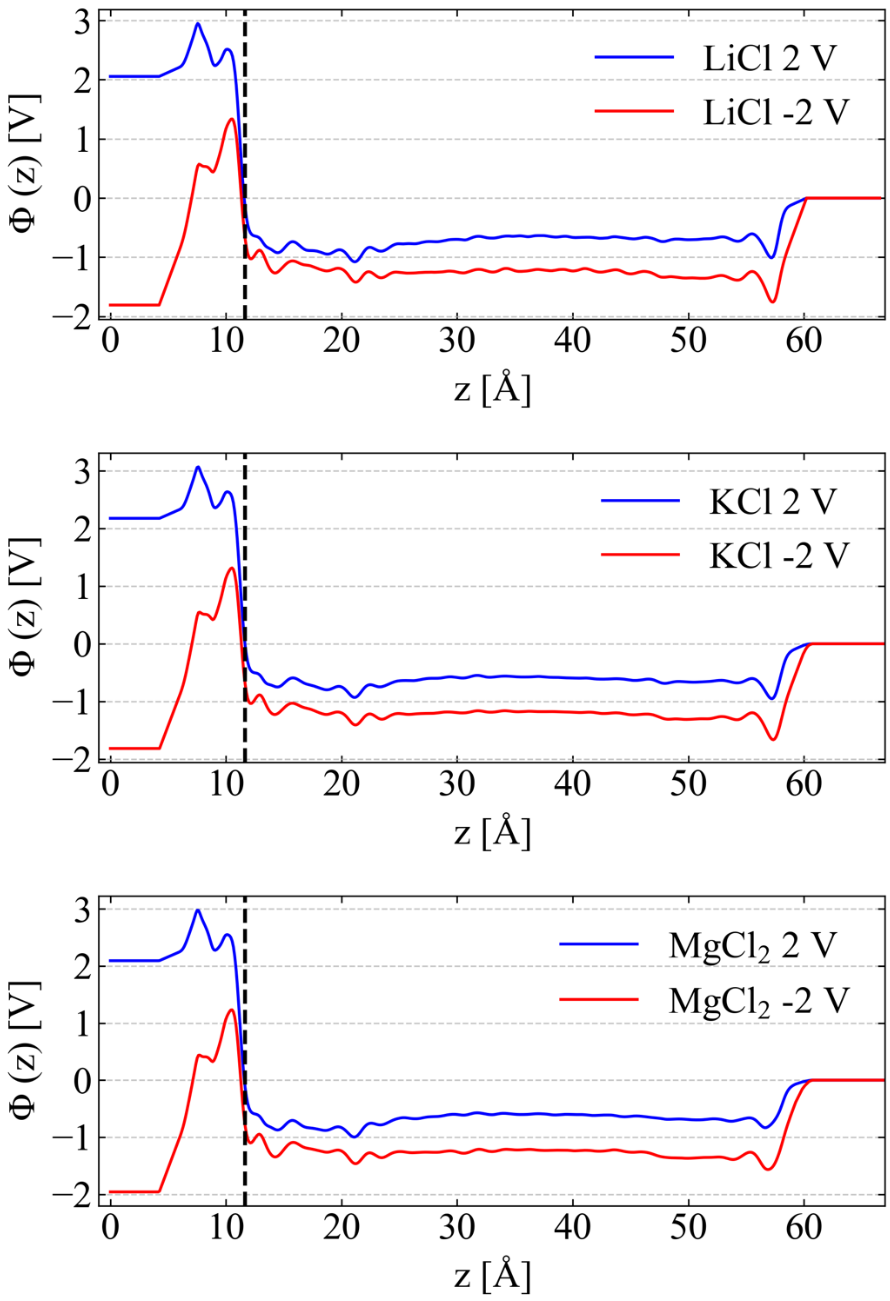


**Figure S21.** Plane-averaged electrostatic potential as a function of $z$-coordinate obtained by integrating the Poisson equation. The vertical dashed line denotes the average position of the N atom of 4-MBN.

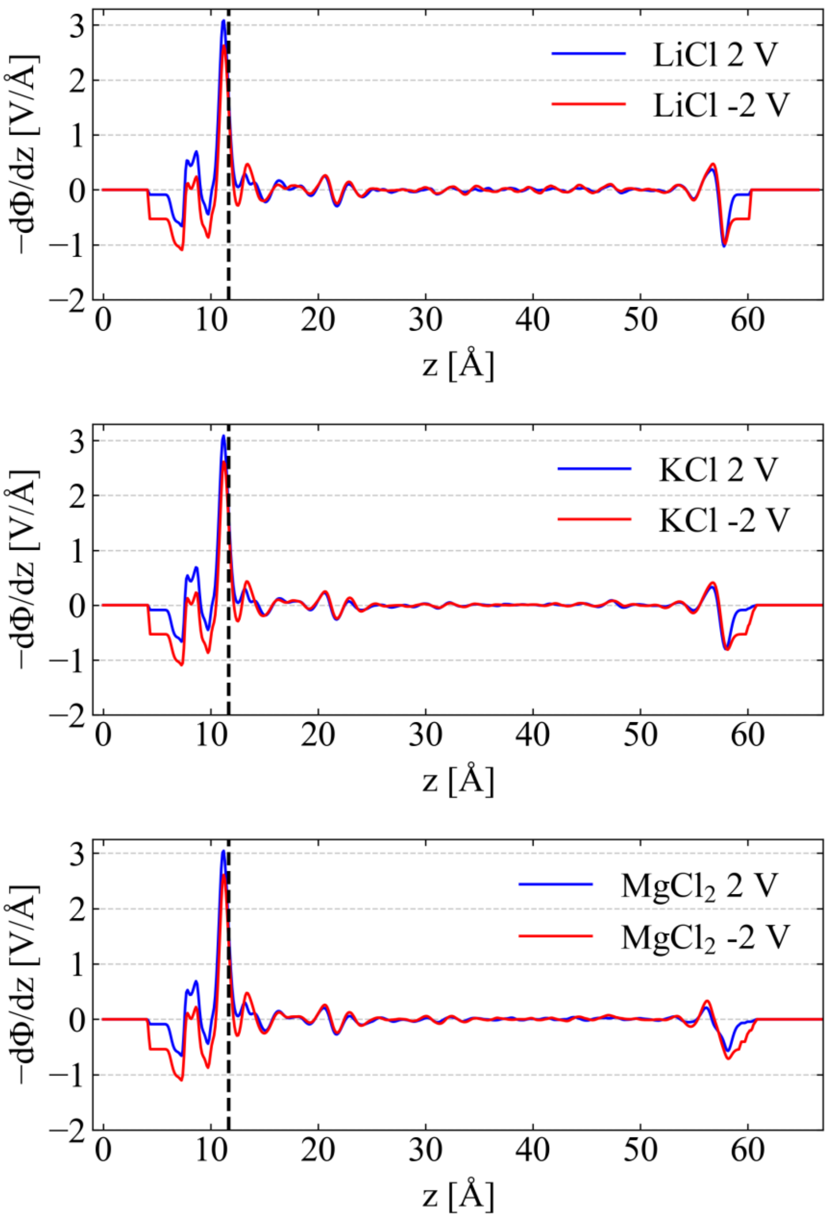


**Figure S22.** Average electric field along the z-direction obtained by differentiating the potential profile with respect to *z*-coordinate. The vertical dashed line denotes the average position of the N atom of 4-MBN.

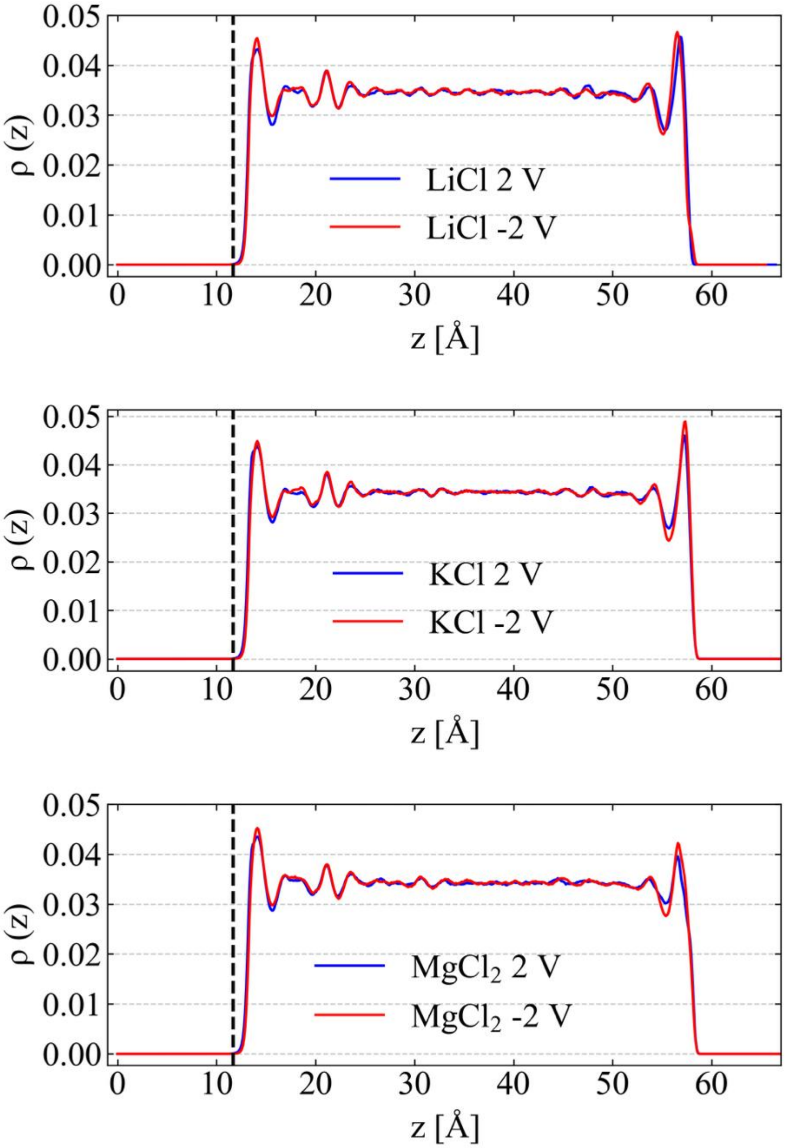


**Figure S23.** Water density as a function of z-coordinate. The vertical dashed line denotes the average position of the N atom of 4-MBN.

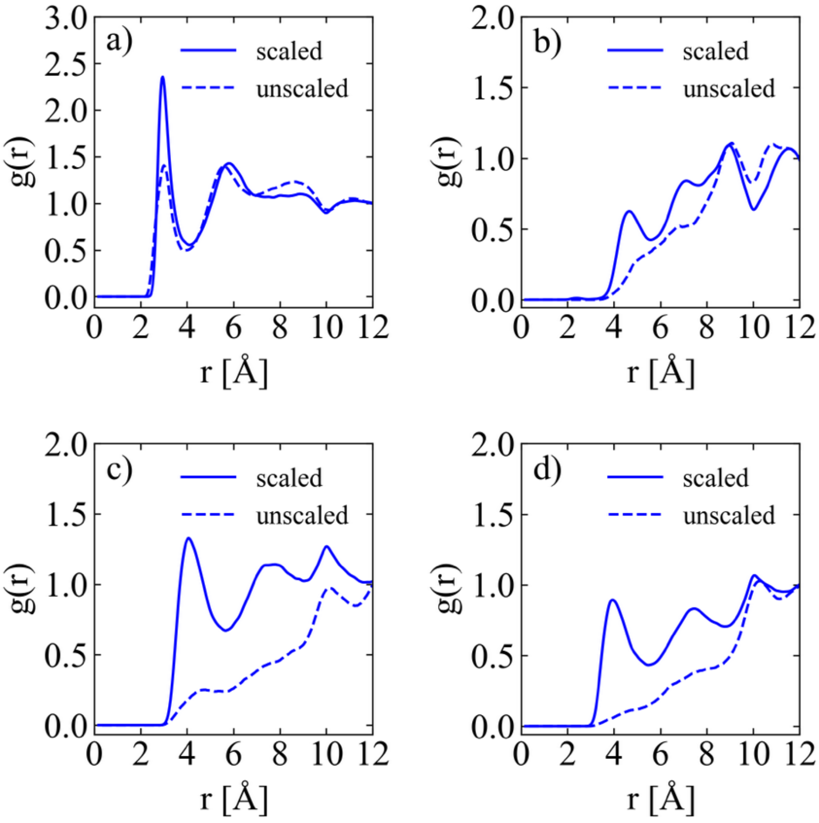


**Figure S24.** Ion-N (4-MBN) radial distribution functions calculated using full ("unscaled") ion charges and scaled charges according to the Electronic Continuum Correction model: a) N-$K^+$ (KCl) at -2V, b) N-$Mg^{2+}$ ($MgCl_2$) at -2V, c) N-$Cl^-$ (KCl) at +2V, and d) N-$Cl^-$ ($MgCl_2$) at 2V.

## References


(1) Duboué-Dijon, E.; Mason, P. E.; Fischer, H. E.; Jungwirth, P., Hydration and Ion Pairing in Aqueous $Mg^{2+}$ and $Zn^{2+}$ Solutions: Force-Field Description Aided by Neutron Scattering Experiments and Ab Initio Molecular Dynamics Simulations. *J. Phys. Chem. B* **2018**, *122*, 3296-3306.
(2) Laage, D.; Stirnemann, G., Effect of Ions on Water Dynamics in Dilute and Concentrated Aqueous Salt Solutions. *J. Phys. Chem. B* **2019**, *123*, 3312-3324.
(3) Ryan, M. J.; Yang, N.; Kwac, K.; Wilhelm, K. B.; Chi, B. K.; Weix, D. J.; Cho, M.; Zanni, M. T., The hydrogen-bonding dynamics of water to a nitrile-functionalized electrode is modulated by voltage according to ultrafast 2D IR spectroscopy. *PNAS* **2023**, *120*, e2314998120.
(4) Yang, N.; Ryan, M. J.; Son, M.; Mavrič, A.; Zanni, M. T., Voltage-Dependent FTIR and 2D Infrared Spectroscopies within the Electric Double Layer Using a Plasmonic and Conductive Electrode. *J. Phys. Chem. B* **2023**, *127*, 2083-2091.
(5) Shim, S.-H.; Zanni, M. T., How to turn your pump–probe instrument into a multidimensional spectrometer: 2D IR and Vis spectroscopiesvia pulse shaping. *PCCP* **2009**, *11*, 748-761.
(6) Kraack, J. P.; Hamm, P., Vibrational ladder-climbing in surface-enhanced, ultrafast infrared spectroscopy. *PCCP* **2016**, *18*, 16088-16093.
(7) Yuan, R.; Fayer, M. D., Dynamics of Water Molecules and Ions in Concentrated Lithium Chloride Solutions Probed with Ultrafast 2D IR Spectroscopy. *J. Phys. Chem. B* **2019**, *123*, 7628-7639.
(8) Kwac, K.; Yang, N.; Ryan, M. J.; Zanni, M. T.; Cho, M., Molecular dynamics simulation study of water structure and dynamics on the gold electrode surface with adsorbed 4-mercaptobenzonitrile. *J. Chem. Phys.* **2024**, *160*, 064701.
(9) Thompson, A. P.; Aktulga, H. M.; Berger, R.; Bolintineanu, D. S.; Brown, W. M.; Crozier, P. S.; in 't Veld, P. J.; Kohlmeyer, A.; Moore, S. G.; Nguyen, T. D.; Shan, R.; Stevens, M. J.; Tranchida, J.; Trott, C.; Plimpton, S. J., LAMMPS - a flexible simulation tool for particle-based materials modeling at the atomic, meso, and continuum scales. *Comput. Phys. Commun.* **2022**, *271*, 108171.
(10) Kwac, K.; Zanni, M. T.; Cho, M., Impact of Electric Field on the Structural Dynamics of Water and Adsorbed Molecules Near an Electrode Surface: Simulations of 2D IR Experiments. *J. Phys. Chem. B* **2025**, *129*, 9495-9505.
(11) Berendsen, H. J. C.; Grigera, J. R.; Straatsma, T. P., The missing term in effective pair potentials. *J. Phys. Chem.* **1987**, *91*, 6269-6271.
(12) Wang, J.; Wang, W.; Kollman, P. A.; Case, D. A., Automatic atom type and bond type perception in molecular mechanical calculations. *J. Mol. Graph. Model.* **2006**, *25*, 247-260.
(13) Wang, J.; Wolf, R. M.; Caldwell, J. W.; Kollman, P. A.; Case, D. A., Development and testing of a general amber force field. *J. Comput. Chem.* **2004**, *25*, 1157-1174.
(14) Zhao, X.; Leng, Y.; Cummings, P. T., Self-Assembly of 1,4-Benzenedithiolate/Tetrahydrofuran on a Gold Surface: A Monte Carlo Simulation Study. *Langmuir* **2006**, *22*, 4116-4124.
(15) Kohagen, M.; Mason, P. E.; Jungwirth, P., Accounting for Electronic Polarization Effects in Aqueous Sodium Chloride via Molecular Dynamics Aided by Neutron Scattering. *J. Phys. Chem. B* **2016**, *120*, 1454-1460.
(16) Leontyev, I.; Stuchebrukhov, A., Accounting for electronic polarization in non-polarizable force fields. *Phys. Chem. Chem. Phys.* **2011**, *13*, 2613-2626.
(17) Mason, P. E.; Wernersson, E.; Jungwirth, P., Accurate Description of Aqueous Carbonate Ions: An Effective Polarization Model Verified by Neutron Scattering. *J. Phys. Chem. B* **2012**, *116*, 8145-8153.
(18) Pluhařová, E.; Mason, P. E.; Jungwirth, P., Ion Pairing in Aqueous Lithium Salt Solutions with Monovalent and Divalent Counter-Anions. *J. Phys. Chem. A* **2013**, *117*, 11766-11773.
(19) Pluhařová, E.; Fischer, H. E.; Mason, P. E.; Jungwirth, P., Hydration of the chloride ion in concentrated aqueous solutions using neutron scattering and molecular dynamics. *Mol. Phys.* **2014**, *112*, 1230-1240.
(20) Rashmi, R.; Balogun, T. O.; Azom, G.; Agnew, H.; Kumar, R.; Paesani, F., Revealing the Water Structure at Neutral and Charged Graphene/Water Interfaces through Quantum Simulations of Sum Frequency Generation Spectra. *ACS Nano* **2025**, *19*, 4876-4886.
(21) Zhu, X.; Riera, M.; Bull-Vulpe, E. F.; Paesani, F., MB-pol(2023): Sub-chemical Accuracy for Water Simulations from the Gas to the Liquid Phase. *J. Chem. Theory Comput.* **2023**, *19*, 3551-3566.
(22) Palos, E.; Bull-Vulpe, E. F.; Zhu, X.; Agnew, H.; Gupta, S.; Saha, S.; Paesani, F., Current Status of the MB-pol Data-Driven Many-Body Potential for Predictive Simulations of Water Across Different Phases. *J. Chem. Theory Comput.* **2024**, *20*, 9269-9289.
